# Immunosuppressive cells in cancer: mechanisms and potential therapeutic targets

**DOI:** 10.1186/s13045-022-01282-8

**Published:** 2022-05-18

**Authors:** Yan Tie, Fan Tang, Yu-quan Wei, Xia-wei Wei

**Affiliations:** 1 Laboratory of Aging Research and Cancer Drug Target, State Key Laboratory of Biotherapy, National Clinical Research Center for Geriatrics of West China Hospital, Sichuan University, No. 37 Guo Xue Xiang, Chengdu, 610041 China; 2 Department of Orthopeadics, Orthopedic Research Institute, West China Hospital, Sichuan University, Chengdu, China

**Keywords:** Immunotherapy, Immunosuppressive cells, Tumor immune microenvironment, Immunosuppressive cellular cytokines

## Abstract

Immunotherapies like the adoptive transfer of gene-engineered T cells and immune checkpoint inhibitors are novel therapeutic modalities for advanced cancers. However, some patients are refractory or resistant to these therapies, and the mechanisms underlying tumor immune resistance have not been fully elucidated. Immunosuppressive cells such as myeloid-derived suppressive cells, tumor-associated macrophages, tumor-associated neutrophils, regulatory T cells (Tregs), and tumor-associated dendritic cells are critical factors correlated with immune resistance. In addition, cytokines and factors secreted by tumor cells or these immunosuppressive cells also mediate the tumor progression and immune escape of cancers. Thus, targeting these immunosuppressive cells and the related signals is the promising therapy to improve the efficacy of immunotherapies and reverse the immune resistance. However, even with certain success in preclinical studies or in some specific types of cancer, large perspectives are unknown for these immunosuppressive cells, and the related therapies have undesirable outcomes for clinical patients. In this review, we comprehensively summarized the phenotype, function, and potential therapeutic targets of these immunosuppressive cells in the tumor microenvironment.

## Background

Immunotherapies have achieved rapid development recently and are promising therapeutic options for cancers, especially advanced diseases. These novel therapies include immune checkpoint inhibitors (ICIs), cellular immune therapies like adoptive transfer of engineered T cells, cancer vaccines, and oncolytic virus. However, the immunosuppressive microenvironment mediated or induced by cancers largely limited the efficacy of these novel immunotherapies [1, 2]. Generally, this immunosuppressive microenvironment is composed of cellular and soluble components, promoting tumor progression and favoring the immune escape of cancers [3, 4]. Innate immune cells such as myeloid-derived suppressor cells (MDSCs), tumor-associated macrophages (TAMs), tumor-associated neutrophils (TANs), tumor-associated dendritic cells (tDCs), and adoptive immune cells like the regulatory T cells (Tregs) are the main cellular components within the tumor microenvironment (TME). Besides, small molecules such as vascular endothelial growth factor (VEGF), transforming growth factor-beta (TGF-β), and cytokines like interleukin-10 (IL-10) released by cancer cells or immunosuppressive cells are proved to be involved in this process [5–7]. In addition, cancer cells also decrease the expression of neoantigens and antigen presentation molecules, such as MHC-I, or upregulate the expression of immune checkpoints on immunosuppressive cells to escape the immune recognition [8, 9]. Thus, therapeutic strategies targeting these immunosuppressive cells include remodeling the TME and increasing the anti-tumor efficacy of immunotherapies. However, even achieved certain success in preclinical studies, some therapies or targets presented limited effectiveness in clinical patients. Thus, in this review, we comprehensively review the current knowledge of the immunosuppressive cells, mainly including MDSCs, TAMs, TANs, Tregs, and DCs in the tumor-associated immunosuppression. We also address the potential strategies to target these immunosuppressive cells for optimizing the therapeutic efficacy in cancer patients.

### The immunosuppressive cells in TME

#### Myeloid-derived suppressive cells (MDSCs)

MDSCs are immature myeloid cells deriving from hematopoietic stem cells residing in the bone marrow. The precursor of MDSCs migrates out of the bone marrow and travels into the extramedullary sites, induced by factors, and becomes the MDSCs. MDSCs are distinctive from neutrophils because they reduce the expression of CD16 and CD62L and increase the expression of Arg-1, CD66B, and CD11b [10]. Currently, several subtypes of MDSCs have been categorized, and each of them has an immunological function. Generally, monocytic MDSCs (M-MDSCs) and polymorphonuclear MDSCs (PMN-MDSCs) are the two main subtypes. M-MDSCs are marked by CD11b^hi^, Ly6C^hi^, and Ly6G^lo^. Instead, PMN-MDSCs are distinguished by a CD11b^hi^, Ly6G^hi^, and Ly6C^lo^ phenotype, while early-stage MDSCs (eMDSCs) display a CD11b^hi^, CD14^−^, CD15^−^, and CD33^−^ phenotype in human [11, 12]. Both M-MDSCs and PMN-MDSCs tend to have an enhanced suppressive phenotype in the TME when compared with conventional MDSCs in peripheral lymphoid organs [13]. M-MDSCs present non-specific suppression mediated by diverse mechanisms such as the secretion of anti-inflammatory and inhibitory cytokines (IL-10, TGF-β, and ROS), the expression of iNOS and Arg-1, and the expression of immune checkpoint inhibitors, and collaboration with other immune cells like Th17 and Tregs. They also produce cytokines and soluble factors that support tumor angiogenesis [14]. PMN-MDSCs are antigen-specific T cell tolerance and non-specific suppression and produce cytokines supporting the angiogenesis in the tumor. eMDSCs are present in peripheral blood as free cells and the TME as enriched cell populations.

Factors responsible for the presentation of MDSCs in TME can be classified into three groups: growth factors including G-CSF, M-CSF, and GM-CSF that are essential for the expansion of MDSCs; cytokines including IL-1 families, IL-4, IL-6, IL-13, and TNF that are inducing the functional maturation of MDSCs; and chemo-attractants including IL-8, CCL2, and CXCL12 for mobilization of MDSCs into TME [15, 16]. After presenting in the TME, MDSCs modulated the interactions between immune cells and the cancer cells, resulting in immunosuppression [17]. Besides, the epithelial–mesenchymal transition (EMT) transcriptional factors, including Snail and Twist1, attracted the immunosuppressive cells such as MDSCs and promoted the expression of immunosuppressive checkpoints, leading to the immunosuppressive TME. In turn, immunosuppressive factors induced tumor cells' EMT, which further promoted the tumor progression [18]. MDSCs are crucial for the pre-metastatic niche formation, which can persist in distant organs (such as the lung) for up to 2 weeks after primary surgeries. These persistent postsurgical MDSCs in the lung showed more robust immunosuppression function than presurgical MDSCs [19, 20]. Overall, due to their critical roles in mediating immunosuppression, MDSCs are regarded as potential targets or biomarkers for immunotherapies [21].

In the clinic, the increase in MDSCs related to tumor progression, attenuated effectiveness of immunotherapies, and poorer outcomes [22, 23]. For example, the results of a whole blood nine colors and 11-parameter flow cytometric assay demonstrated significantly higher levels of MDSC among patients with hepatocellular carcinoma than age-matched healthy controls [24]. Thus, detection of the peripheral blood M-MDSC may be used as a prognostic marker in the clinic [25]. The blood concentration of MDSCs could be good prognostic markers for overall survival after 2 years in refractory diffuse large B-cell lymphoma [21]. In addition, tumors responding to ICIs therapy tend to have higher CD8^+^ T cells and fewer Gr-1^+^CD11b^+^ MDSCs at the early stage following therapy initiation in the mouse model [26]. Furthermore, in patients with different cancer types who received ICIs, the circulating and tumor-infiltrating myeloid populations can be used as predictive biomarkers, both at baseline and on treatment [27].

#### Tumor-associated macrophages (TAMs)

TAMs are proposed to be the most abundant cell type in TME, orchestrating the immunosuppressive microenvironment [28]. TAMs are usually recruited via chemokines action from the periphery as monocytes and then settled in tumor tissues. Tissue-resident macrophages also migrate to hypoxic or necrotic areas in tumors and then are induced to become TAMs. The number of tumor-infiltrating TAMs is related to poor prognosis in most cancer patients, such as breast cancer, hepatoma, lung cancer, gastric cancer, and other malignancies [29, 30]. Inflammatory M1-macrophages (classically activated) and immunosuppressive M2-macrophages (alternatively activated) are the two main sub-types of macrophages [31]. M1-macrophages are marked with CD11b^+^F4/80^+^CD206^−^, and CD11c^+^, and M2-macrophages tend to be CD11b^+^F4/80^+^CD206^+^ in mouse model (Table 1) [31, 32]. Different stimulating factors induce the phenotypes of macrophages. For example, LPS is essential for M1-macrophages; IL-4, IL-10, IL-13 for M2a-macrophages; TLR agonists for M2b-macrophages; TNFα, glucocorticoids for M2c-macrophages; and TLR and adenosine A2A receptors for M2d-macrophages [33]. More markers are utilized for the phenotype of macrophages [17]. Besides, the functional mechanisms of each type of macrophage are different. M1-macrophages prompted the destruction of tumor cells, recruited tumor-killing leukocytes, and engulfed tumor cells by producing the immune-killing molecules such as ROS and nitrogen and inflammatory cytokines such as IL-6 and TNF-α. However, M2-macrophages are demonstrated to express co-inhibitory molecules, such as PD-L1, and release anti-inflammatory cytokines, such as IL-10 [34]. In addition, M2-type TAMs enhanced the tumor progression and orchestrated the tumor development and metastasis by releasing matrix metalloproteinases (MMPs), raking down the basement membrane, and remodeling the epithelial cell movements, inducing angiogenesis by releasing VEGF, and recruiting Tregs and MDSCs [28, 35]. Based on this mechanism, dual inhibition of TAMs and PMN-MDSCs is proved to potentiate the efficacy of ICIs [36].

**Table 1:** Phenotypes and functions of immunosuppressive cells within the TME

Cell type	Sub-type	Phenotypes	Functions
MDSCs [11, 12]	M-MDSCs	Mouse: CD11b^+^Ly6C^high^Ly6G^−^ Human: CD11b^+^CD14^+^CD33^+^HLA-DR^−^CD15^−^ CD11b^+^CD14^+^CD33^+^HLA-DR^−^CD15^+^	Induce Non-specific suppression; Produce cytokines that support tumor angiogenesis; Induce anti-tumor therapy resistance
	PMN-MDSCs	Mouse: CD11b^+^Ly6G^+^Ly6C^low^ Human: CD11b^+^CD14^−^CD15^+^CD33^+^HLA-DR^−^ CD11b^+^CD14^−^CD66b^+^	Induce antigen-specific T cell tolerance and non-specific suppression; Produce cytokines that support tumor angiogenesis
	eMDSCs	Human: CD11b^+^CD14^−^CD15^−^CD33^+^HLA-DR^−^	Exist as free cells in the peripheral blood and as enriched cell populations in the tumor microenvironment
Macrophages [28]	M1	Mouse: CD11b^+^F4/80^+^CD206^−^ Human: CD64^+^CD80^+^	Produce immune killing molecules; Secrete specific inflammatory cytokines; Display pro-inflammatory features
	M2	Mouse: CD11b^+^F4/80^+^CD206^+^ Human: CD163^+^CD86^+^	Remodel the ECM; Brake down the basement membrane; Promote angiogenesis; Construct immunosuppression; Recruit Tregs and MDSCs; Orchestrate tumor development and distant metastasis
Neutrophils [37, 38]	N1	Mouse: CD11b^+^Ly6G^+^CD54^+^CD16^+^CD170^low^, CD177^+^ (in CRC) Human: CD11b^+^CD66b^+^CD101^+^CD54^+^HLA-DR^+^CD86^+^CD15^high^CD170^low^, CD177^+^ (in CRC)	Associate with anti-tumor properties; Characterized by a normal density, a hypersegmented nucleus and a cytotoxic activity toward cancer cells
	N2	Mouse: CD11b^+^Ly6G^+^PDL1^+^CD170^high^ Human: CD11b^+^CD66b^+^PDL1^+^CD170^high^	Associated with pro-tumor properties; Have immunosuppressive activity
	N_I_	Mouse: CD11b^+^Ly6G^+^CD117^+^CD170^low^CD101^−^CD84^+^JAML^+^ Human: CD11b^+^CD66b^+^CD117^+^CD10^−^CD16^int/low^LOX1^+^CD84^+^JAML^+^	Immature neutrophils endow with immunosuppressive properties appear in the circulation, primary tumors and metastases
	N_ISG_	Mouse: CD11b^+^Ly6G^+^, IFIT1, IRF7, RSAD2 Human: CD11b^+^CD66b^+^, IFIT1, IRF7, RSAD2	Neutrophils with interferon-stimulated gene signatures
Tumor-associated DCs [68, 69, 72]	Plasmacytoid DCs	Mouse: B220^+^CD11c^low^MHC-II^+^CD303^+^ Human: B220^+^CD11c^low^MHC-II^+^CD317^+^	Contribute to tumor-induced immunosuppression
	Conventional DCs	Mouse: Lin^−^ZBTB46^+^MHC-II^+^CD141^+^; Lin^−^ZBTB46^+^MHC-II^+^CD11c^+^; CD11b^+^	Induce Th2 responses; Suppress CD8^+^ T cell function
	Inflammatory DCs	Mouse: CD11c^+^MHC-II^+^CD11b^+^F4/80^+^Ly6C^+^CD206^+^CD115^+^CD107b^+^FcɛRI^+^CD64^+^ Human: CD11c^+^CD115^+^CD1c^+^CD1a^+^FcɛRI^+^CD206^+^CD172a^+^CD14^+^CD11b^+^	Produce high levels of pro-inflammatory cytokines, such as TNF, IL-6, and IL-12
Tregs [52–54]	nTregs	CD4^+^CD25^+^Foxp3^+^CD127^lo/−^	Maintain normal immune tolerance and control the inflammatory response
	iTregs	CD4^+^CD25^+^Foxp3^+^CD127^lo/−^	Inhibit the anti-tumor immune action of effector T cells, NK cells and DCs; Secrete inhibitory cytokines; Kill effector cells by granzymes and perforin; Produce factors to facilitate Tregs expansion and reinforce the suppressive environment
Bregs [72, 78, 79]	B10 cells	Mouse: CD5^+^CD1d^hi^ Human: CD19^+^CD24^hi^CD27^+^	Produce IL-10, and suppress effector CD4^+^ T cells, monocytes, and DCs; Induce Tregs through TGF-β
	T2-MZP cells	Mouse: CD19^+^CD21^hi^; CD23^hi^CD24^hi^ Human: CD19^+^CD24^+^CD38^+^	Produce IL-10, and suppress effector CD4^+^ T cells; Induce Tregs and decrease CD8^+^ T cells by TGF-β

#### Tumor-associated neutrophils (TANs)

The differentiation of granulocyte-monocyte progenitors into neutrophils is associated with mediators expressed by G-CSF and GM-CSF and starts with the formation of myeloblasts. Then, the myeloblasts differentiate into promyelocytes. The promyelocytes subsequently produce the myelocytes, metamyelocytes, and mature neutrophils [37, 38]. The phenotype of neutrophils in TME depends on the type and stage of cancer. Neutrophils are pro-inflammatory in the early stage of tumor initiation, while they adapt to be an immunosuppressive type as the tumor progresses [17, 39–41]. TANs have N1 (anti-tumor) and N2 (tumor-promoting) phenotypes: N1-TANs are marked by CD11b^+^Ly6G^+^CD54^+^CD16^+^CD170^low^ and CD177^+^ in mice; However, N2-TANs is characterized by CD11b^+^Ly6G^+^PD-L1^+^CD170^high^. Another classification strategy is based on the expression of selected molecules, including N_I_, N_M_, N_A,_ and N_ISG,_ referring to the immature neutrophils, mature neutrophils, aged neutrophils, and neutrophils with interferon-stimulated gene signatures, respectively (Table 1) [37].

TANs suppress the T cells' activation and promote genetic instability, angiogenesis, and tumor metastasis [37, 42]. For mechanisms, TANs inhibit the activation of T cells through secreting the Arg-1, ROS, and NO induced by G-CSF and TGF-β [12, 43]. Besides, TANs promote the proliferation of tumor cells via the production of growth factors like EGF, HGF, and PDGF [44, 45]. The release of neutrophil extracellular traps (NETs) containing HMGB1, neutrophil elastase, myeloperoxidase, and matrix metalloproteinases (MMP8/9) and activating the integrin signaling also promotes the proliferation of tumor cells [46]. NETs are comprised of MMPs, cathepsin G neutrophil elastase, and myeloperoxidase which induces the production of pro-inflammatory cytokines and orchestrates the TME, thereby enhancing tumor progression and metastasis [47, 48]. Prostaglandin E2 (PGE2), expressed by infiltrating neutrophils, promoted tumor proliferation via regulating the inflammation-related gene expression in a self-amplification manner [49]. Besides, TANs also promote tumor angiogenesis by releasing the pro-angiogenic factors BV8, S100A8/9, and MMPs that activate VEGFA in the extracellular matrix [37, 50]. Neutrophil-derived oncostatin M induced the VEGF from cancer cells and increased the detachment and invasive activity of cancer cells [51]. Inhibition of TGF-β signals drove neutrophils to switch to an N1 phenotype with anti-tumor properties. In a TGF-β-rich microenvironment, neutrophils typically have N2 profiles with properties promoting tumor progression and immunosuppressive function [37].

#### Regulatory T cells (Tregs)

Tregs universally labeled by CD4^+^CD25^+^Foxp3^+^CD127^low/−^ [52–54] are differentiated from traditional T cells and divided into two sub-groups, including naturally occurring Tregs (nTregs) and induced-to-adjust T cells (iTregs). These two types of Tregs commonly express the classic marker, Foxp3 [53, 55, 56]. nTregs naturally expand in the normal thymus, and they acquire an inhibitory effect via intercellular interaction. Based on this evidence, nTregs are also called thymus-derived Tregs (tTregs). nTregs regulate the inflammatory response and preserve normal immune tolerance, which can be activated and maintained by NF-κB [57]. iTregs are supposed to be differentiated from peripheral naive T cells induced by factors in TME, including the tumor antigens, inhibitory cytokines such as TGF-β, and other soluble molecules. iTregs are also named peripherally induced Tregs (pTregs) in vivo. iTregs inhibit the anti-tumor immune response of effector T cells, NK cells, and DCs, resulting in tumor progression [53]. There is a new strategy for Tregs classification in which Th-like Tregs subsets were characterized to elaborate their tissue distribution and biological function. For more details, Th1-like Tregs are marked as T-bet^+^IFN-γ^+^Foxp3^+^, Th2-like Tregs are labeled with Gata3^+^IRF4^+^IL-4^+^Foxp3^+^, and Th17-like Tregs are marked as IL-17^+^RORγt^+^Foxp3^+^ [53]. Th2-like Tregs expressed Gata3 and IRF4 and secret the cytokines IL-4 and IL-13. Th2-like Tregs were induced by IL-4R signaling, which promoted the Gata3 expression [58]. Th17-like Tregs are supposed to express Foxp3 and RORγt, which can be differentiated in the periphery from conventional T cells that retain the suppressive function [59].

Tregs are a double-edged sword by regulating immune homeostasis and inhibiting cancer immune responses. IFN-γ secreted by Tregs enhanced the efficacy of ICIs [60]. The intra-tumoral suppressing T effector cells contributed to the cancer progression that is associated with poor prognosis [61, 62]. Tregs secreted inhibitory cytokines, including IL-10 and TGF-β, thereby inhibiting the immune function [63]. For example, Tregs inhibited the function of CD8^+^ T cells and DCs through membrane-bound TGF-β [64, 65]. In addition, Tregs eliminated the effector cells by granzymes and perforin, which mediate the cytotoxicity of T lymphocytes and NK cells [53, 66]. Tregs also play essential roles in the angiogenesis through VEGF/VEGFR pathway. It reported that IL-2 on Tregs regulates the effector CD8^+^ T cells, and the loss of IL-2 would partly explain the exhausted phenotype occurring during tumor responses. This evidence suggests that Tregs influence the function of effector cells by interfering with cell metabolism [67].

#### Tumor-associated dendritic cells (tDCs)

DCs are differentiated from hematopoietic stem cells, which are resident in the bone marrow. Tumor-inducing signals prevent the production of mature, immunologically active DCs and, at the same time, transform tumor-associated DCs (tDCs) from immunostimulatory cell types to immunosuppressive cell types [68, 69]. This immunosuppressive tDCs included diverse subtypes because of their high heterogeneity, including plasmacytoid DCs (pDCs), conventional DCs (cDCs), and monocyte-derived inflammatory DCs (moDCs) marked by different immune markers (Table 1) [68]. tDCs suppress the activation of cytotoxic T cells and promote the generation of immunosuppressive Tregs, which further regulate the immunostimulatory phenotype at an early stage of tumor progression to an immunosuppressive phenotype [70, 71]. tDCs inhibit the excitation of cytotoxic CD8^+^ T cells by expressing or secreting immune inhibitory molecules such as PD-L1, T cell immunoglobulin and mucin domain 3 (TIM-3), VEGF, IL-10, PGE-2, and TGF-β [72]. In addition, DCs also promoted the instability of the genome and the angiogenesis to support tumor plasticity [73, 74]. pDCs enhance immunosuppression by promoting the secretion of IL-10 from CD4^+^Foxp3^−^ T cells [75]. cDCs stimulated the Th-2, Th-17, and T regulatory responses [76]. At last, the moDCs were derived from monocytes and produced pro-inflammatory cytokines, including TNF, IL-12, and IL-6, to promote tumor progression [77].

#### Other immunosuppressive cells

**Fig. 1:**
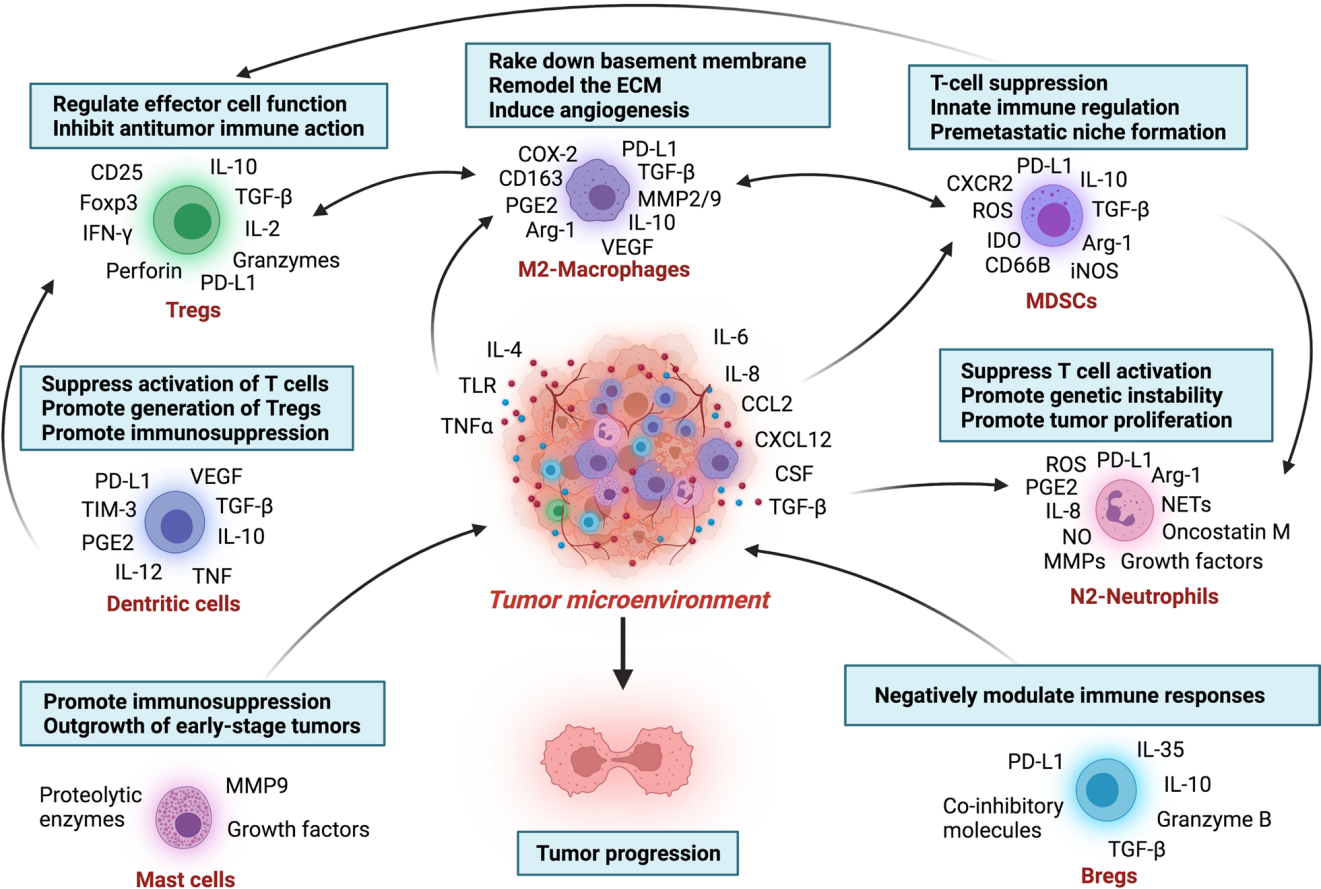
The immunosuppressive cells in tumor micro-environment. Immune cells infiltrate into the tumor microenvironment, interact with each other and tumor cells, and then harbor an immunosuppressive phenotype that is responsible for the immune escape of tumor cells and the following tumor progression. These immunosuppressive cells include MDSCs, M2-macrophages, Tregs, N2-TANs, mast cells, Bregs, dendritic cells. They secrete cytokines like IL-2, IL-10, and TGF-β, growth factors like VEGF, the checkpoints ligands like PD-L1 or express checkpoints on the cell surface like PD-1, TIM-3 on Tregs, that negatively regulate the anti-tumor immune response, remodel the extracellular matrix, and promote the angiogenesis. As a result, these immunosuppressive cells and their interaction generate an immunosuppressive microenvironment and promote the proliferation, evasion, and migration of tumor cells

Besides the above immunosuppressive cells, other immune cells existed in TME, mediating the immunosuppressive TME described in recent years. For example, B cells play opposing roles in cancers by suppressing the anti-tumor immunity, of which Bregs are associated with inflammation, autoimmunity, and tumor progression [78, 79]. Bregs negatively modulated the immune responses by releasing anti-inflammatory cytokines, such as IL-10, and expressing co-inhibitory molecules, such as PD-L1 [80, 81]. Another immune cell involved in tumor progression is the tumor-associated mast cell. Mast cells are promoters, bystanders, or guardians of tumors following the cancer stage. Mast cells are essential for the outgrowth of early-stage prostate cancer due to the secretion of MMP9 but become dispensable in advanced-stage [82]. Activated tumor-associated mast cells induced immunosuppression, angiogenesis, tumor invasion, and metastasis by releasing growth factors and proteolytic enzymes [83]. Besides, it has been demonstrated that the number of infiltrating mast cells within the tumor is related to poor prognosis in cancer patients (Fig. 1).

### Targeting immunosuppressive cells in cancers

#### MDSC-based therapies

**Fig. 2:**
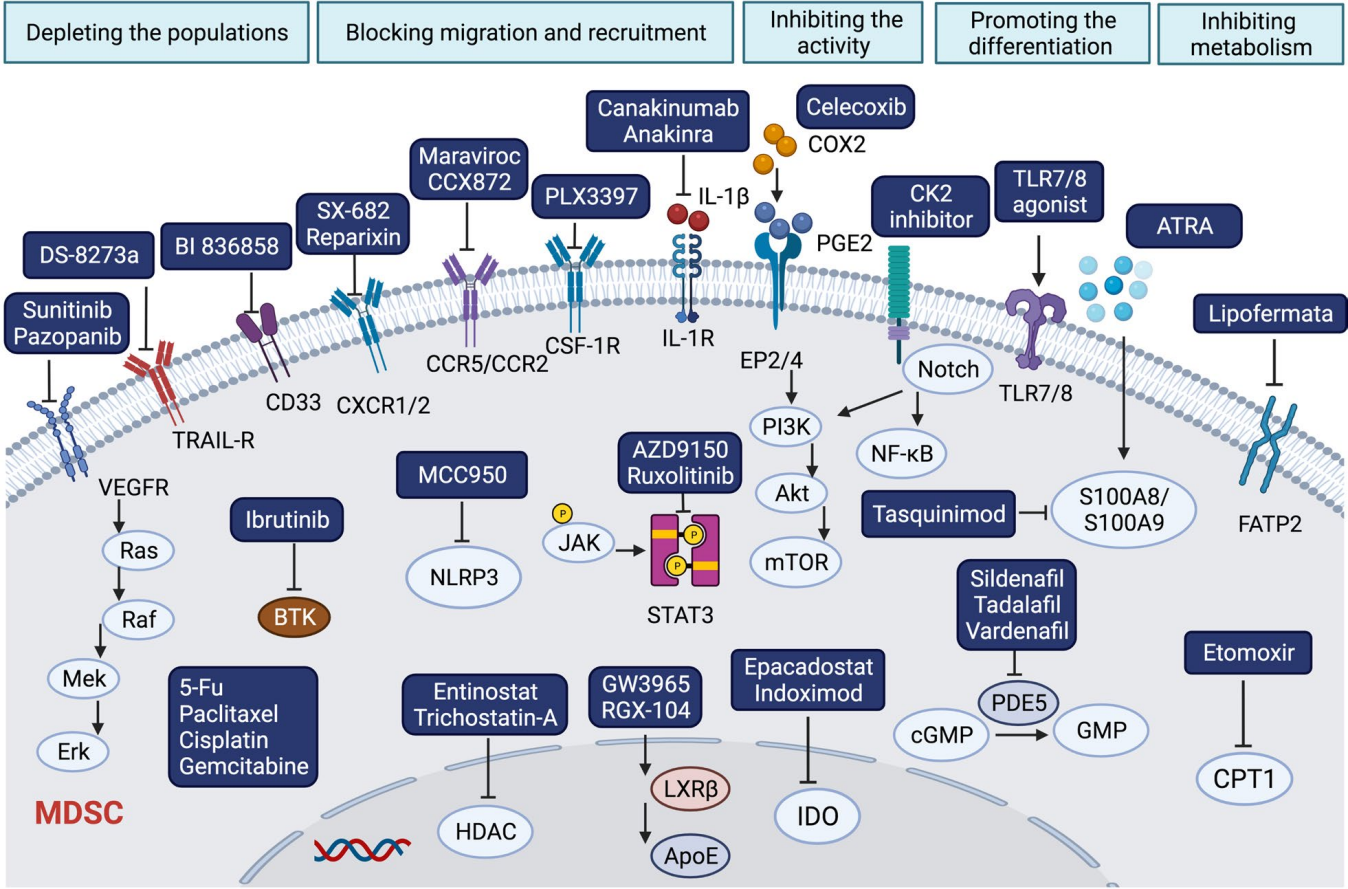
The potential strategies to target MDSCs. MDSC is the main type of immunosuppressive cell in cancer. Strategies targeting MDSCs to reverse the immunosuppression include depleting the populations of MDSCs by targeting VEGFR and CD33, blocking the migration and recruitment of MDSCs into TME by targeting the CCR2 or CXCR1/2, inhibiting the activity of MDSCs by targeting PGE2 and IDO, promoting the differentiation of MDSCs by TLR agonists, and inhibiting the metabolism of MDSCs by targeting FATP2 and CPT1

Strategies targeting MDSCs include depleting the populations, blocking their migration and recruitment, inhibiting their activity, inhibiting MDSCs metabolism, and promoting the maturation of MDSCs [84]. Here, we summarized the targets involved in the therapeutic strategies based on MDSCs for cancer therapy (Fig. 2).

#### Depleting the populations of MDSCs

Low-dose chemotherapeutics, including paclitaxel, cisplatin, 5-fluorouracil (5-FU), and gemcitabine, effectively deplete MDSC populations and enhance anti-tumor immune activity [85–88]. Moreover, targeting the signals involved in MDSC expansion inhibited the proliferation of MDSCs. Tyrosine kinase inhibitors (TKIs) are extensively studied to eliminate MDSCs. For example, sunitinib prevented the accumulation of MDSCs and restored the T cells' function in renal carcinoma cancer, colorectal cancer, and breast cancer-bearing mice via blockade of VEGF and STAT3 signaling [89]. The inhibitor of Bruton's tyrosine kinase (BTK), ibrutinib, was reported to inhibit the generation and modulate the function of MDSCs in mammary tumors and melanoma to enhance immune-based therapy [90]. Besides, the administration of pazopanib resulted in a dramatic decrease in MDSC and the following enhancement of the PD-1 expressing cytotoxic T and NK effectors (Table 2) [91]. The effect of chemotherapeutics on MDSCs depends on various factors, including chemotherapy doses, dosing schedules, tumor types and stages, and the location of MDSCs. Chemotherapeutics are not specific to MDSCs and affect all rapidly proliferating cells, including anti-tumor T cells. Therefore, the effect of chemotherapy on tumor immunity depends on the balance between immunostimulatory and immunosuppressive effects.

**Table 2:** Ongoing clinical trials to target MDSCs in cancer patients

Target	Agent	Tumor types and combinational therapies	Phase	Sample size	Clinical trial number
CXCR2 inhibitor	AZD5069	Metastatic Castration Resistant Prostate Cancer + Enzalutamide	I/II	86	NCT03177187
	SX-682	Pancreatic Cancer + Nivolumab	I	20	NCT04477343
		Melanoma + Pembrolizumab	I	77	NCT03161431
CCR5 inhibitor	Vicriviroc	Advanced/Metastatic Microsatellite Stable Colorectal Cancer + Pembrolizumab	II	42	NCT03631407
	Maraviroc	Advanced Metastatic Colorectal and Pancreatic Cancer + Nivolumab + Ipilimumab	I	50	NCT04721301
CCR5 antibody	Leronlimab	Locally Advanced or Metastatic Solid Tumors	II	30	NCT04504942
STAT3 inhibitor	OPB-31121	Advanced Cancers	I	24	NCT00955812
	AZD9150	Advanced Solid Tumors + Durvalumab	I	76	NCT03421353
	WP1066	Recurrent Malignant Glioma and Brain Metastasis from Melanoma	I	33	NCT01904123
	TTI-101	Advanced Cancers	I	60	NCT03195699
	SC-43	Advanced NSCLC and Advanced Biliary Tract Cancer + Cisplatin	I/II	100	NCT04733521
	OPB-51602	Advanced Cancer	I	45	NCT01423903
JAK/STAT3 inhibitor	Ruxolitinib	Head and Neck Squamous Cell Carcinoma	II	45	NCT03153982
PDE5 inhibitor	Tadalafil	Head and Neck Squamous Cell Carcinoma	II	40	NCT01697800
	Sildenafil	Solid Tumor + Regorafenib	I	32	NCT02466802
COX2 inhibitor	Celecoxib	Lung Cancer + Radiation Therapy	I/II	21	NCT00046839
		Colon Carcinoma + Nivolumab/Ipilimumab	II	60	NCT03026140
		Breast Cancer	III	2639	NCT02429427
		Cervix Neoplasms	I/II	31	NCT00152828
		Endometrium Cancer	II	48	NCT03896113
HDAC inhibitor	Entinostat	Metastatic Colorectal Cancer + Azacitidine	II	47	NCT01105377
	Vorinostat	Locally Advanced NSCLC + Chemotherapy and Radiation Therapy	I	18	NCT01059552
		Malignant Solid Tumor + Hydroxychloroquine	I	72	NCT01023737
	Quisinostat	NSCLC/Epithelial Ovarian Cancer + Chemotherapy	I	51	NCT02728492
	SB939	Prostate Cancer	II	32	NCT01075308
	Panobinostat	Melanoma + Ipilimumab	I	17	NCT02032810
	Chidamide	Cervical Cancer + Toripalimab	I/II	40	NCT04651127
	CHR-3996	Solid Tumor	I	40	NCT00697879
Vitamins	ATRA	Breast Cancer + Anastrozole	II	112	NCT04113863
		Pancreatic Cancer + Chemotherapy	II	170	NCT04241276
		Prostate Cancer + 5-Azacitidine + Lupron	II	20	NCT03572387
		Melanoma + Pembrolizumab	I/II	26	NCT03200847
Casein kinase inhibitor	Silmitasertib (CX 4945)	Advanced Basal Cell Carcinoma	I	26	NCT03897036
		Cholangiocarcinoma + Chemotherapy	I/II	127	NCT02128282
Chemotherapy	Low-Dose Capecitabine + Bevacizumab	Glioblastoma	I	12	NCT02669173
LXR Agonist	RGX-104	Malignant Neoplasms + Immunotherapy/Chemotherapy	I	135	NCT02922764
Tyrosine kinase inhibitor	Pazopanib	Solid Tumors	II	57	NCT01956669
		Solid Tumors + Topotecan	I/II	66	NCT02303028
		Sarcoma + Durvalumab	II	37	NCT03798106
	Ibrutinib	Malignant Solid Tumors + Nivolumab	I	15	NCT03525925
		Lung Cancer	I/II	13	NCT02321540
		Gastroesophageal Cancer	II	17	NCT02884453

DS-8273a, a TNF-related apoptosis-induced ligand receptor (TRAIL-R2) agonistic antibody, reduced the levels of MDSCs in circulation and decreased tumor-infiltrating MDSCs in advanced cancer patients (NCT02076451) [92, 93]. BI 836858 is a humanized Fc-engineered monoclonal antibody targeting CD33 and depleting the MDSCs through the antibody-dependent cellular cytotoxicity [94]. Besides, the immunotoxin gemtuzumab ozogamicin, a humanized monoclonal antibody against CD33, decreased the level of MDSCs and restored the CAR-T cell effects against cancers [95]. In addition, other researchers have developed a novel peptide‐Fc fusion protein therapeutically targeting the S100A family proteins, thereby depleting MDSCs without affecting the pro-inflammatory immune cells [96]. These strategies are under evaluation in preclinical and clinical studies, and further studies are required to investigate the potential roles in tumors.

#### Blockade of MDSCs migration and recruitment

The chemokines of CXCR2, like CXCL2 or CXCL5, are essential for PMN-MDSCs recruitment. Targeting the chemokine receptor CXCR2 reduced the MDSCs populations, promoted T cells' infiltration, and improved the efficacy of PD-1 blockade [97, 98]. SX-682, a CXCR1/2 inhibitor, enhanced the T cell-based immunotherapeutic efficacy and benefited patients with MDSC-infiltrated cancers [99, 100]. Besides, CXCR1/2 agonists are the primary mediators of cancer-promoted NETosis [101]. Other CXCR1/2 inhibitors such as reparixin, navarixin, and AZD5069 have also been evaluated in clinical trials to treat patients with advanced cancer [102]. HuMax-IL8 (BMS-986253), a humanized mAbs targeting IL-8, has been assessed to be tolerable when combined with nivolumab in patients with advanced solid tumors in phase I/II study (NCT03400332) [103, 104]. Besides, the combination of CXCR1/2 inhibitors with chemotherapy, anti-angiogenesis drugs, and immunotherapy is also a prospective strategy in cancer therapy.

Another chemokine receptor is CCR5, regulating the migration and recruitment of M-MDSCs into the TME. In addition, MDSCs expressing CCR5 are more immunosuppressive than MDSCs do not express CCR5. Besides, CCR5/CCR5 ligand axis also activates their immunosuppressive functions in the TME, with therapeutic potential [105]. In preclinical studies, silencing of host CCL5 expression in bone marrow delivered by nanoparticles combined with CCR5 inhibitors (Maraviroc) reduced MDSCs and increased anti-tumor immunity [106]. For clinical patients, the blockade of CCR5 inhibits the recruitment and immunosuppressive function of MDSCs, which improves the overall survival of melanoma and breast cancer [105, 107]. In KR158 glioma-bearing animals, the CCR2 antagonist (CCX872) increased the median survival when administrated as monotherapy and further prolonged overall survival when combined with immunotherapy. Further evaluation of the TME indicated that CCX872 treatment decreased tumor-associated MDSCs, elevated tumor-infiltrating lymphocytes, and increased IFN-γ expression [108].

CSF-1/CSF-1R axis promoted the differentiation of MDSCs following the tumor progression [109]. Inhibition of CSF-1/CSF-1R signaling reduced the tumor-infiltrating MDSCs and enhanced the anti-tumor T cell responses [109]. Thus, treatment with CSF-1R inhibitors disrupted the interaction between carcinoma-associated fibroblasts and MDSCs, triggering an increase in the recruitment of granulocytes to the tumors. The CSF-1R inhibitor and CXCR2 antagonist combination blocked tumor granulocyte infiltration and presented strong anti-tumor efficacy [110]. Improved effects are also observed when CSF-1/CSF-1R blockade combines with anti-VEGFR antibodies and ICIs in vivo [111, 112]. Combination therapies based on CSF-1R blockade require further exploration as promising strategies in cancer patients [84].

Other strategies blocking the migration and recruitment of MDSCs include anti-VEGF/VEGFR therapy, anti-S100A8/A9 therapy, and anti-IL-1β therapy in preclinical studies. VEGF is an essential stimulator in the proliferation and recruitment of MDSCs, and the MDSCs, in turn, would promote tumor angiogenesis via secreting cytokines, such as VEGF [113, 114]. Bevacizumab-based therapies significantly reduced the circulating MDSCs levels in patients with solid tumors, especially combining with chemotherapies [115–117]. IL-1β promoted tumor progression by inducing inflammation, increasing the E-selectin expression, and driving the migration of MDSCs [118–120]. Nucleotide oligomerization domain-like receptor family pyrin domain-containing 3 (NLRP3) inflammasome promoted the maturation and secretion of IL-1β. Thus, agents that inhibited the IL-1/IL-1β included IL-1Ra (anakinra), IL-1β antibody (canakinumab), and the inflammasome inhibitor (MCC950) have potential anti-tumor activity in cancers [121, 122]. S100A8/9 are small molecular calcium-binding proteins expressed on MDSCs and involved in tumor progression [123, 124]. Tasquinimod is an oral agent blocking S100A9, reducing the MDSCs infiltration into TME [125, 126]. Currently, several clinical trials have verified that treatment with tasquinimod would improve the free survival of solid cancer patients by reducing the recruitment of MDSCs (NCT01234311, NCT01743469, NCT00560482) [127–129].

#### Inhibiting the activity of MDSCs

Several strategies are reported to reverse the potent immunosuppressive mechanisms of MDSCs to restore the T cell activity and enhance the anti-tumor immunotherapy efficacy. For example, the antisense oligonucleotide STAT3 inhibitor, AZD9150, showed a marked decrease in PMN-MDSCs and is combined with ICIs for diffuse large B‐cell lymphoma (NCT01563302) [130]. Utilizing the TLR9-targeted STAT3 siRNA delivery to block the immunosuppressive function of MDSCs reduced the expression and enzymatic activity of Arg-1, downregulated the STAT3 target genes, and inhibited the activity of T cells [131]. These similar STAT3-dependent mechanisms of MDSCs are also observed in pancreatic cancer [132]. Inhibition of STAT3 potentially enhances CAR-T cells' efficacy administrated in liver metastasis by modulating the MDSCs [133]. In addition, the autocrine of IL-6 mediated activation of the STAT3-DNA methyl transferase axis silenced the TNFα-receptor interacting protein 1 necroptosis pathway that sustained the accumulation of MDSCs. Therefore, targeting IL-6 represents potentially an effective approach to suppressing the survival and accumulation of MDSCs in the TME [134]. JAK-STAT inhibition with ruxolitinib resulted in an anti-tumorigenic TME, characterized by the decreased tumor-promoting cytokines and the reduced infiltrating MDSCs in lung cancer [135].

Phosphodiesterase-5 (PDE5) inhibitors, including sildenafil, vardenafil, and tadalafil, are currently in clinical application for malignant tumors. In tumor mice models, PDE5 inhibition downregulated the Arg-1, and the expression of nitric oxide synthase-2 reduced the recruitment of MDSCs. Besides, the PDE5 inhibition improved the anti-tumor efficacy of adoptive T cells therapy, reversed the tumor-induced immunosuppression, and enabled a favorable anti-tumor immune response that substantially delays tumor progression [136]. In addition, tadalafil reduced the infiltration of MDSCs and Tregs, promoting anti-tumor immunity in patients with head and neck squamous cell carcinoma [137].

PGE2 is crucial for inducing the phenotype and function of MDSCs, whereas the tumor-promoted induction of MDSCs depends on COX2 signaling. The essential role of the COX2-PGE2 pathway in the expansion and persistence of MDSCs highlights the potential function of its management to enhance or suppress immune responses in tumors. Disruption of the COX2-PGE2 axis with COX2 inhibitors suppresses the production of MDSCs-related suppressive factors [138]. In mouse models of glioma, treatment with the COX2 inhibitors, acetylsalicylic acid, or celecoxib inhibited the systemic production of PGE2 and delayed the development of glioma [139]. Besides, targeting the COX2-PGE2 feedback eliminated the self-limiting of type-1 immunity in TME, driven by the synergistic induction of COX2, and enhanced the efficacy of immunotherapies for cancers [140]. The COX2/PGE2 feedback regulates the expression of PD-L1 in tumor-infiltrating myeloid cells. Using COX2 inhibitors to inhibit PGE2 formation reduced PD-L1 expression on TAMs and MDSCs. Therefore, reprogramming the PGE2 metabolism in TME provided a chance to reduce immune suppression [141].

Histone deacetylase inhibitors (HDACs) play essential roles in inducing the differentiation of MDSCs [142]. A combination of low-dose adjuvant epigenetic drugs disrupted the pre-metastatic microenvironment and inhibited the tumor metastases [143]. Low-dose HDAC inhibitor trichostatin-A reshaped the TME and upregulated the expression of PD-L1 by modulating the suppressive activity of TAMs and MDSCs, further enhancing the anti-tumor effects of immunotherapies. Combining low-dose trichostatin-A with anti-PD-L1 significantly enhanced the durable tumor reduction and prolonged the overall survival of tumor-bearing mice [144]. Entinostat enhanced the anti-tumor effect of PD-1 targeting by functionally inhibiting MDSCs and transforming from an immunosuppressive microenvironment [145]. Combining entinostat with anti-PD-1 or anti-CTLA-4 significantly decreased the suppression by PMN-MDSCs, increased in activated granzyme-B producing CD8^+^ T cells, and improved the tumor-free survival in both breast cancer and pancreatic cancer [146]. Therefore, further research is required to explore the mechanism of combining immunotherapy with HDAC and to develop effective therapies for cancer patients.

Other potential strategies inhibiting the function of MDSCs are also explored and evaluated. For example, the nuclear factor erythroid 2-related factor 2 (Nrf2) pathway was a potential therapeutic target in the tumor, the activation of which cleared the ROS in MDSCs and decreased the risk of tumor metastasis [147, 148]. Besides, the synthetic triterpenoid CDDO-Me inhibited the immunosuppressive activity of MDSCs via activating Nrf2 and inhibiting the generation of ROS in MDSCs (NCT00529113) [149]. In addition, the NOV-002, a glutathione disulfide mimetic, induced the S-glutathionylation and inhibited the production of ROS in MDSCs (NCT00499122) [150, 151].

#### Promoting the differentiation and maturation of MDSCs

All‐trans‐retinoic acid (ATRA) is supposed to induce the differentiation of MDSCs into mature myeloid cells. ATRA induced the differentiation of MDSCs primarily through the neutralization of high ROS generation, involving a specific increase in the glutathione synthase and the accumulation of glutathione [152]. ATRA treatment blocked the anti-angiogenic therapy-induced expansion of MDSC. Concomitant therapy with ATRA holds the potential to improve anti-angiogenic therapy in breast cancer [153]. Due to the function of modulating the differentiation of MDSCs, the combinational therapies of ATRA and immunotherapies have synergistic anti-tumor efficacy. For example, ATRA combined with ipilimumab significantly reduced the number of MDSCs in peripheral circulation compared with ipilimumab monotherapy in patients with metastatic melanoma (NCT02403778) [154]. In addition, combinational therapy utilizing GD2-CAR T cells and ATRA significantly improved the anti-tumor efficacy against sarcoma in a mouse model. Retinoids can reduce the suppressive function of MDSCs, while co-administration of retinoids can enhance the effectiveness of CAR T cells therapy in solid tumors [155]. The combination of anti-PD-1 therapy with ATRA improved the proliferation of local and systemic T cells and produced tumor-specific immunity. Accumulation of MDSCs in LKB1-deficient NSCLC can be overcome via the administration of ATRA, sensitizing the tumors to immunotherapy [156]. Overall, ATRA is an effective strategy in anti-tumor therapies, while off-target effects may contribute to diminished therapeutic efficacy. ATRA can enhance immune response via inducing the differentiation of MDSCs, especially in combination with other anti-tumor strategies.

The increased activity of casein kinase 2 (CK2) is responsible for the Notch phosphorylation and downregulation. Utilizing siRNA or pharmacological inhibitors to inhibit CK2 restored the Notch signaling in myeloid cells and significantly improved their differentiation [157]. Besides, inhibition of CK2 substantially reduced the amount of PMN-MDSCs by a block of differentiation. These modulatory effects of CK2 inhibitors on myeloid cell differentiation in the TME would be synergized with the anti-tumor effects of ICIs [158].

Other potential therapies included promoting the differentiation and maturation of MDSCs. For example, the TLR7/8 agonists enhanced the anti-tumor efficacy by promoting the differentiation of MDSCs into macrophages and DCs (NCT02124850) [159, 160]. Besides, polyinosinic–polycytidylic acid, namely poly I: C, which is a synthetic double-stranded RNA ligand for TLR3, has shown promising potential in decreasing the MSDCs and abrogating their immunosuppressive functions, especially when combined with irradiation, vaccines, and CAR-T therapy [161–163]. Finally, the traditional Chinese medicine saposhnikovia root divaricate prim-o-glucosylcimifugin was identified as an inhibitor of PMN-MDSCs. The prim-o-glucosylcimifugin exhibited a favorable synergistic anti-tumor efficacy with a PD-1 inhibitor [164]. Also, the administration of β-glucans, curdlan, curcumin, and icariin accelerated the differentiation of MDSCs and weakened the related immunosuppression in preclinical tumor models [165–168]. Nevertheless, these strategies in cancer patients require further investigation.

#### Inhibiting MDSCs metabolism

Transcription factors liver-X nuclear receptors (LXRβ and LXRα) are associated with the fatty acid, cholesterol, glucose, and lipid metabolism. These receptors are potential targets for regulating the metabolism of MDSCs. For example, LXR agonists, GW3965 and RGX-104, reduced the accumulation of MDSCs in mice models. In a phase I trial, patients were treated with first-in-human dose-escalation by targeting the LXR/apolipoprotein E axis to regulate innate immune suppression and activation of cytotoxic T lymphocyte (CTL) responses [169]. LXR agonist upregulated the apolipoprotein E (ApoE), which binding to the low-density lipoprotein receptor-related protein 8 (LRP8) expressed on MDSCs, leading to the depletion of MDSCs [169, 170]. Another fatty acid-related target is carnitine palmitoyltransferase 1 (CPT1, a rate-limiting enzyme in the fatty acid pathway). Etomoxir, a CPT1 inhibitor, significantly abrogated the immunosuppressive function of MDSCs and delayed the tumor growth when combined with low-dose chemotherapy [171]. Lipofermata, a fatty acid transport protein 2 (FATP2) inhibitor, restrained the activity of PMN-MSDCs and restricted the tumor progression through the STAT5 pathway alone or combined with ICIs [172, 173].

Glycolysis and the related pathways are potential targets to modulate the immunosuppressive activity of tumor-infiltrating MDSCs. The glycolysis of MDSCs in cancers is modulated by HIF-1α and AMP-activated protein kinase (AMPK) [174, 175]. Metformin activated the AMPK, thereby inhibiting the immunosuppressive function of MDSCs and delaying the tumor progression by inhibiting immune-related NF-κB and JAK-STAT pathways [174, 176, 177].

Indoleamine 2,3-dioxygenase (IDO) is a critical enzyme in the tryptophan–kynurenine–aryl hydrocarbon receptor axis. IDO is over-expressed in tumor-infiltrating immune cells and tumor cells, facilitating the recruitment and activation of MDSCs [178–180]. The cytokine IL-6 triggered the IDO promotor in MDSCs through the STAT3 pathway [181]. A phase III study involving the IDO inhibitor, epacadostat, combined with pembrolizumab, has comparable outcomes between the epacadostat group and placebo group in patients with advanced melanoma (NCT02752074) [182]. Recently, another phase II trial of indoximod showed that the addition of indoximod to a taxane did not improve the PFS compared with a taxane alone in patients with ERBB2-negative metastatic breast cancer (NCT01792050) [183]. Finally, novel inhibitors targeting the Trp-Kyn-AhR pathways, such as navoximod, tryptophan mimetics, kynurenine-degrading enzymes, and aryl hydrocarbon receptor antagonists, are also explored in cancer therapy based on MDSCs [182, 184–186].

### The TAMs-based targeted therapies

**Fig. 3:**
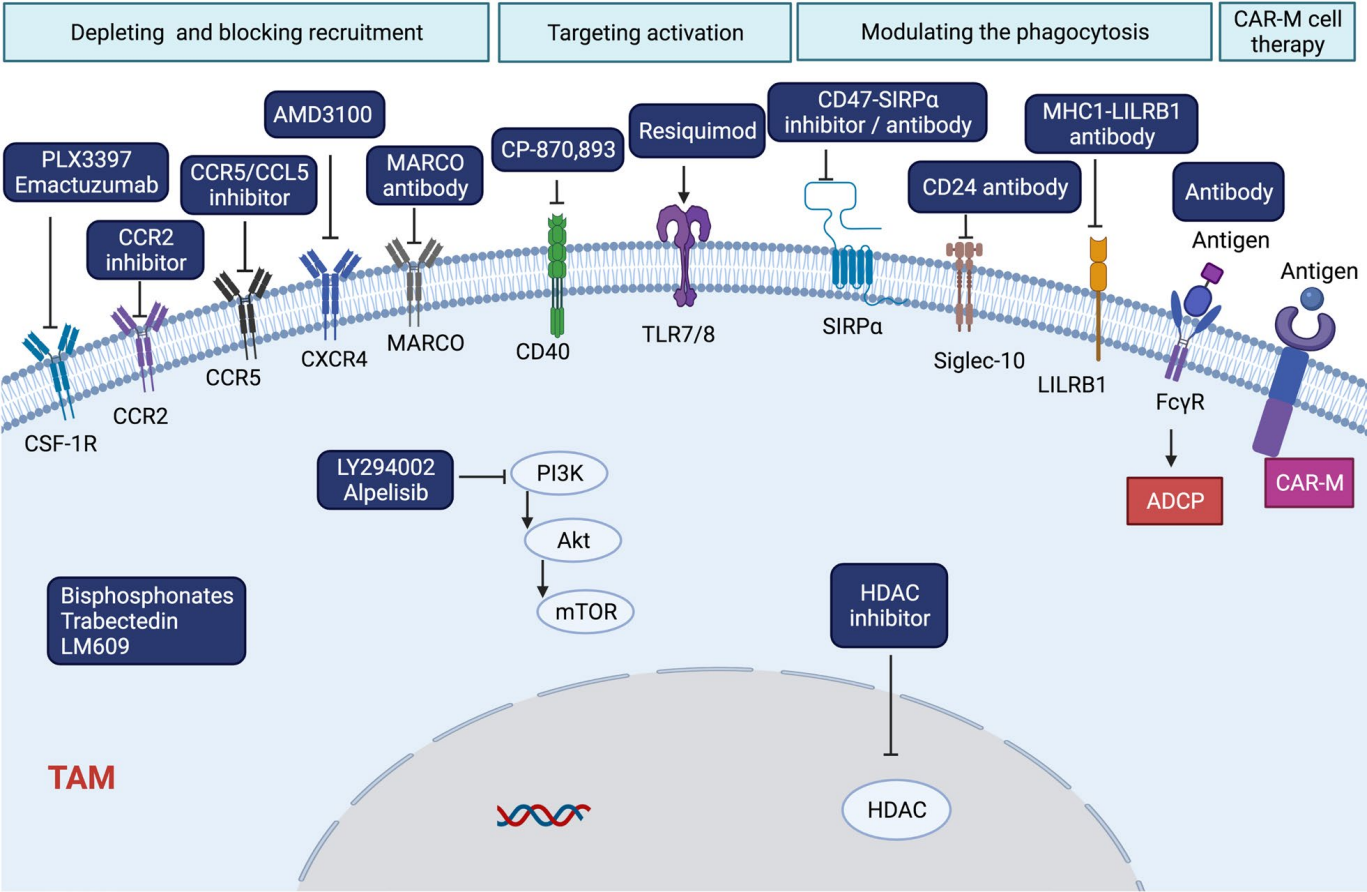
The potential targets of TAMs in cancer therapy. M2-type TAMs in TME mediate the immunosuppression and promote the growth of tumor cells, as well as the resistance of cancer to immunotherapy. Strategies targeting the TAMs to reverse the immunosuppression include depleting and blocking the recruitment of TAMs into TME by targeting the CCR2 or CXCR1/2, targeting the activation of TAMs by CD40 and TLR7/8, modulating the phagocytosis of TAMs by targeting SIRPα, LILRB1, and Siglec-10. Furthermore, with the development of adoptive cell therapy, CAR-M represents a novel strategy that applies modified macrophages by adding specific CAR to them, which enhances the phagocytosis of macrophages on tumor cells. Besides, other advantages of CAR-M are identified, such as the resistance of CAR-M being polarized to M2 type

Strategies targeting TAMs include depleting macrophages and blocking the recruitment of TAMs, targeting the activation of TAMs, modulating the phagocytosis of TAMs, and chimeric antigen receptor macrophage cell therapy. Here, we concluded the relevant therapeutic strategies on TAMs for cancer therapy (Fig. 3).

#### Depleting TAMs and blocking the recruitment of TAMs

CSF-1R is expressed on macrophages and their frontier monocytes, which is an important signal for the differentiation of TAMs. Nanoparticles loaded with anti-CSF-1R siRNA specifically depleted the M2-TAMs from melanoma and then restored the immune function of T cells, decreased the tumor size, and prolonged the overall survival [187]. 3D185 is a novel double inhibitor targeting both the FGFRs and CSF-1R. This inhibitor inhibited the polarization of macrophages to M2-like and the tumor progression by remodeling the tumor microenvironment in TAM-dominated murine models [188]. PLX3397, also named pexidartinib, is a CSF-1R kinase inhibitor that is supposed to reduce the numbers of TAMs effectively and the angiogenesis and ascites in mouse models with mesothelioma. PLX3397 combined with DC vaccination synergistically enhanced the overall survival with decreased TAMs and increased the number and function of CD8^+^ T cells [189]. A clinical trial suggested that emactuzumab, a monoclonal antibody against CSF-1R, combined with paclitaxel, depletes immunosuppressive TAMs in patients with advanced solid tumors [190]. In addition, CSF-1R inhibition altered the polarization of macrophages via secreting GM-CSF and IFN-γ, which blocked glioma progression in a mouse model [191]. Furthermore, targeting TAMs using a CSF-1R inhibitor combined with radiotherapy enhanced the survival of glioma in preclinical models (Table 3) [192]. These therapeutic strategies are not necessarily effective because CSF-1/CSF1R blockade may increase the activity of Tregs in the TME and compensate by signaling through other pro-survival pathways.

**Table 3:** Ongoing clinical trials to target TAMs in cancer patients

Target	Agent	Tumor types and combinational therapies	Phase	Sample size	Clinical trial number
CSF1-R inhibitor	Pexidartinib (PLX3397)	Metastatic Breast Cancer + Eribulin	Ib/II	67	NCT01596751
		Prostate Adenocarcinoma + Radiation Therapy + Antihormone Therapy	I	8	NCT02472275
		Metastatic/Advanced Pancreatic or Colorectal Cancers + Durvalumab	I	48	NCT02777710
		Pigmented Villonodular Synovitis/Tenosynovial Giant Cell Tumor	III	120	NCT02371369
	DCC-3014	Sarcoma + Avelumab	I	48	NCT04242238
		Advanced Tumors and Tenosynovial Giant Cell Tumor	I/II	120	NCT03069469
	ARRY-382	Advanced Solid Tumors + Pembrolizumab	II	82	NCT02880371
		Metastatic Cancer	I	26	NCT01316822
	SNDX-6352	Unresectable Intrahepatic Cholangiocarcinoma + Durvalumab	II	30	NCT04301778
		Solid Tumor + Durvalumab	I	45	NCT03238027
	BLZ945	Advanced Solid Tumors + PDR001	I/II	200	NCT02829723
CSF1-R antibody	Cabiralizumab (BMS-986227)	Peripheral T Cell Lymphoma + Nivolumab	II	33	NCT03927105
		Advanced Solid Tumors + Nivolumab	I	295	NCT02526017
		Solid Tumors + Nivolumab + APX005M	I	120	NCT03502330
		Pigmented Villonodular Synovitis/Diffuse Type Tenosynovial Giant Cell Tumor	I/II	75	NCT02471716
	IMC-CS4 (LY3022855)	Solid Tumor	I	72	NCT01346358
		Solid Tumor + Durvalumab/Tremelimumab	I	178	NCT02718911
		Pancreatic Cancer + Cyclophosphamide + Pembrolizumab	I	12	NCT03153410
	RO5509554/Emactuzumab/RG7155	Advanced Solid Tumors	I	217	NCT01494688
VEGFR/Aurora B/CSF1-R inhibitor	Chiauranib	Hepatocellular Carcinoma	I/II	27	NCT03245190
		Small Cell Lung Cancer	I	27	NCT03216343
		Ovarian Cancer	I/II	25	NCT03166891
VEGFR/KIT/RET/BRAF/CSF1-R inhibitor	Regorafenib	Hepatocellular Carcinoma + Nivolumab	I/II	60	NCT04170556
		Hepatocellular Carcinoma	II	171	NCT04476329
VEGFR/FGFR/PDGFRβ/Kit/RET/TrkA/FLT3/CSF1-R inhibitor	Dovitinib (TKI258, Novartis)	Gastrointestinal Stromal Tumors	II	30	NCT01440959
MET/CSF1-R/SRC inhibitor	TPX-0022	Advanced Solid Tumors with MET mutation	I	120	NCT03993873
AXL/MER/CSF1-R inhibitor	Q702	Solid Tumor	I	78	NCT04648254
CCL2 antibody	CNTO888	Solid Tumors + Gemcitabine/Docetaxel/Paclitaxel and Carboplatin	I	53	NCT01204996
CCR2 antibody	MLN1202	Metastatic Cancer	II	44	NCT01015560
CCR2/5 inhibitor	BMS-813160	NSCLC/HCC + Nivolumab	II	50	NCT04123379
		Pancreatic Ductal Adenocarcinoma + Gemcitabine + Nab-paclitaxel + Nivolumab	I/II	39	NCT03496662
CXCR4 inhibitor	BL-8040	Metastatic Pancreatic Adenocarcinoma + Pembrolizumab	II	23	NCT02907099
	Plerixafor (Mozobil)	Advanced Pancreatic, Ovarian and Colorectal Adenocarcinomas	I	26	NCT02179970
		Metastatic Pancreatic Cancer + Cemiplimab	II	21	NCT04177810
Bisphosphonates	Zoledronic Acid	Hormone-Refractory Prostate Cancer	II	30	NCT00636740
		Bone Metastatic NSCLC	II	60	NCT04325776
	Ibandronic Acid	Breast Cancer	II	171	NCT02616744
	Alendronate Sodium	Breast Cancer	III	303	NCT00122356
Trabectedin	Trabectedin	Liposarcoma/Leiomyosarcoma	II	105	NCT02247544
		Soft Tissue Sarcoma + Olaratumab	I	28	NCT03985722
		Ovarian and Uterine Carcinosarcoma	II	45	NCT02993705
		Malignant Pleural Mesothelioma	II	145	NCT02194231
		Metastatic Adult Soft Tissue Sarcoma + Nivolumab	II	92	NCT03590210
		Pancreatic Cancer	II	25	NCT01339754
		Advanced Soft Tissue Sarcomas	II	132	NCT00003939
TLR 4 agonist	GLA-SE	Adult Soft Tissue Sarcoma	I	16	NCT02180698
		Merkel Cell Carcinoma	I	10	NCT02035657
	GSK1795091	Cancer	I	42	NCT02798978
TLR 7/8 agonist	TransCon TLR7/8 Agonist	Solid Tumor + Pembrolizumab	I/II	140	NCT04799054
	BDB018	Solid Tumor + Pembrolizumab	I	50	NCT04840394
	NKTR-262	Solid Tumor	I/II	64	NCT03435640
TLR 7 agonist	RO7119929	Hepatocellular Carcinoma, Biliary Tract Cancer, Solid Tumors With Hepatic Metastases	I	100	NCT04338685
	Imiquimod	Breast Cancer	II	10	NCT00899574
TLR 9 agonist	SD-101	Pancreatic Adenocarcinoma + Nivolumab + Radiation Therapy	I	6	NCT04050085
		Solid Tumor + BMS 986,178	I	12	NCT03831295
	CMP-001	Melanoma + Pembrolizumab	II	54	NCT04708418
		Pancreatic Adenocarcinoma and Melanoma + INCAGN01949	I/II	42	NCT04387071
	IMO-2125 (Tilsotolimod)	Refractory Melanoma + Ipilimumab	III	454	NCT03445533
		Malignant Melanoma	II	214	NCT04126876
	EMD 1,201,081	Recurrent or Metastatic Squamous Cell Carcinoma of the Head and Neck + Cetuximab	II	107	NCT01040832
CD40 agonist antibody	Selicrelumab (RO7009789)	Solid Tumors + Atezolizumab	I	140	NCT02304393
	RO7300490	Solid Tumors + Atezolizumab	I	280	NCT04857138
	LVGN7409	Cancer	I	126	NCT04635995
	CP-870,893	Melanoma + Tremelimumab	I	25	NCT01103635
	Mitazalimab	Metastatic Pancreatic Ductal Adenocarcinoma + Chemotherapy	I/II	70	NCT04888312
	CDX-1140	Breast Cancer + CDX-301, Radiotherapy, and Poly-ICLC	I	36	NCT04616248
	APX005M	Neoadjuvant Therapy for Rectal Cancer + Chemotherapy	II	58	NCT04130854
Anti-CD47 antibody	ZL-1201	Advanced Cancer	I	66	NCT04257617
	IBI188	Advanced Malignancies	I	92	NCT03717103
	Hu5F9-G4	Solid Tumor	I	88	NCT02216409
	STI-6643	Solid Tumor	I	24	NCT04900519
	AK117	Neoplasms Malignant	I	162	NCT04728334
	ALX148	Head and Neck Cancer + Pembrolizumab	II	111	NCT04675294
	AO-176	Solid Tumor	I/II	183	NCT03834948
Anti-CD47/PD-1 bispecific antibody	HX009	Advanced Solid Tumor	II	210	NCT04886271
		Advanced Solid Tumor	I	21	NCT04097769
	PF-07257876	Advanced or Metastatic Tumors	I	90	NCT04881045
	IBI322	Advanced Solid Tumor	I	36	NCT04912466
		Advanced Solid Tumor	I	45	NCT04338659
		Advanced Solid Tumor	I	218	NCT04328831
PI3Kγ inhibitor	IPI-549	Head and Neck Squamous Cell Carcinoma	II	15	NCT03795610
Modified Vitamin D Binding Protein Macrophage Activator	EF-022	Solid Tumors	I	24	NCT02052492
Migration Inhibitory Factor (MIF)	Anti-MIF Antibody	Malignant Solid Tumors	I	68	NCT01765790

CCL2/CCR2 axis is associated with several types of immunosuppressive cell recruiting, including TAMs, MDSCs, and Tregs [193–195]. Targeting TAMs by inhibiting CCR2 decreased the tumor-initiating cells, resulting in metastasis inhibition and enhancement of the anti-tumor T cell responses in pancreatic tumor and liver cancer [196–198]. Dual targeting of the CCR2^+^ TAMs and CXCR2^+^ TANs improved the anti-tumor immunity in pancreatic adenocarcinoma [199]. CCL2/CCR2 inhibitors, while having some success in preclinical models, have not demonstrated similar levels of efficacy in clinical trials. CCL2/CCR2 blockade has little effect on established TAMs and still promotes tumor progression. Besides, upregulation of stromal cell-derived factor 1 alpha (SDF-1α/CXCL12) induced by hypoxia was supposed to contribute to the recruitment of TAMs [200, 201]. AMD3100, a CXCR4 antagonist, prevented the polarization of TAMs toward an immunosuppressive phenotype after the administration of sorafenib, which inhibited tumor growth and reduced the lung metastases in mice models [202]. Besides, inhibiting CXCR4 combined with anti-angiogenesis drugs improved the anti-tumor efficacy via the ERK pathway in liver cancer [203].

Bisphosphonates depleted TAMs that suppressed the angiogenesis, normalized the tumor vasculatures, and relieved tumor hypoxia, which offered favorable synergistic therapeutic efficacy [204–206]. Nanoparticles containing zoledronic acid caused a significant reduction of the TAMs, inducing the complete remission and increasing the survival of tumor xenografts [207]. The same preclinical outcome was observed in the S180 tumor-bearing mice model administrated with Bletilla striata polysaccharide and alendronate conjugate [208]. Yondelis, also named Trabectedin, is a novel therapeutic agent extracted from the tunicate Ecteinascidia turbinate, resulting in inhibition of the viability and function of TAMs [209]. Renalase is a secreted flavoprotein that increased markedly in TAMs, promoting melanoma progression. Therefore, targeting renalase has potential therapeutic implications for the management of melanoma [210]. Besides, the accumulation of TAMs is related to the expression of integrin αvβ3 on tumor cells, which are known drivers of epithelial tumor progression and drug resistance. LM609 is a monoclonal antibody targeting αvβ3, and this agent engages the macrophages to induce cellular cytotoxicities on αvβ3-expressing tumor cells [211].

#### Targeting the activation of TAMs

Reprogramming TAMs toward the M1 phenotype would prevent immunosuppression and improve anti-tumor immunity. The selective depletion of the microRNA processing enzyme DICER in TAMs promoted M1-like macrophages programming, characterized by overactive IFN-γ/STAT1 signaling. DICER/Let-7 activity against IFN-γ-induced immunostimulatory M1-like TAM activation has potential therapeutic significance [212]. Agonistic anti-murine TNF receptor such as CP-870893, a fully human anti-CD40 monoclonal antibody, has been assessed in clinical trials with beneficial efficacy in melanoma and pancreatic cancers [213, 214]. The combination therapy of agonistic anti-CD40 monoclonal antibody and CSF-1R inhibitor effectively inhibits tumor growth in mouse models of melanoma by targeting TAMs, transforming “cold” into “inflamed” tumor microenvironment [215]. TAM programming with agonistic anti-CD40 increased both Ly6C^high^ TAMs and the intra-tumoral accumulation of TCR-engineered T cells, which finally increased tumor cell apoptosis [216].

TAM activation inhibited the TLR-mediated M1-type macrophage polarization. Treatment with resiquimod, a TLR7/8 agonist, doubled the overall survival in mice models [217]. R848-Ad, an adamantane-modified derivative of resiquimod, enables TAM-targeted drug delivery and then enhances the anti-tumor effects in a mice colon cancer model [218]. In addition, targeted delivery of the long peptide antigen to TAMs through nanohydrogels with a TLR agonist activated the TAMs, inducing antigen presentation activity and then transforming resistant tumors into susceptibility to adoptive transfer of tumor-specific T cell receptor engineered T cells [219]. Combining the TLR agonists with focal ablation drives a highly effective immune response [220].

The PI3K/AKT pathway sustains the recruitment of inflammatory cells and is essential for tumor invasion. LY294002, a PI3Kα inhibitor, blocked the recruitment of TAMs, in addition to inhibit the TAM-mediated tumor invasion [221]. Another PI3Kα inhibitor, alpelisib (BYL719), had favorable anti-tumor activity in head and neck squamous cell carcinoma. But the TYRO3 and AXL receptors meditated the resistance to alpelisib in this model [222]. Combined treatment with PI3Kα and either EGFR, AXL, or PKC inhibitors reverted this resistance [223, 224]. In addition, PIK3CA mutations predicted the response to PI3K inhibitors in lung cancer. The combination of PI3K inhibitor and CDK4/6 inhibition enhanced the anti-tumor response to the two single agents [225].

#### Modulating the phagocytosis of TAMs

Signal regulatory protein alpha (SIRPα) on TAMs interacts with CD47, a “don't eat me” signal on cancer cells, preventing the phagocytosis of macrophages for cancer cells [226]. Thus, disruption of the SIRPα-CD47 axis would protect effectively against solid tumors by inducing tumor phagocytosis [227–229]. In pancreatic cancers, targeting CD47 efficiently enhanced the phagocytosis of tumor cells and the apoptosis of macrophages [230]. Efficiently blockade of the CD47-SIRPα pathway with genetically engineered cell-membrane-coated magnetic nanoparticles promoted the repolarization of M2-TAMs, triggering the potent immune responses in melanoma and breast cancer models [231, 232]. Currently, the anti-CD47 monoclonal antibodies, including Hu5F9-G4, CC-9002, TI-061, and SRF231, are under clinical trial evaluation [233]. CD47/SIRPα blockade cannot induce phagocytosis on its own, which should be used in combination with an opsonizing agent.

CD24 is suggested as a novel “don't eat me” signal that promotes the tumor immune escape [234, 235].CD24 is a dominant innate immune checkpoint regarding as a promising immunotherapy target for cancer. CD24 interacting with the inhibitory receptor sialic-acid-binding Ig-like lectin 10 (Siglec-10) on TAMs promoted the evasion of the tumor. Gene ablation of CD24 or Siglec-10 and monoclonal antibodies to block the CD24-Siglec-10 interaction enhanced the phagocytosis of CD24-expressing tumors, resulting in a decrease in macrophage-dependent tumor growth [236, 237].

MHC-I controls the phagocytic function of macrophages, mediated by the inhibitory receptor LILRB1 on TAMs. Disruption of MHC-I or LILRB1 potentiated the macrophages' phagocytosis of cancer cells, which defined the MHCI-LILRB1 pathway axis as an “eat me/don't eat me” signal [238]. Antibody-dependent cellular phagocytosis (ADCP) is an essential mechanism for antibody tumor therapy based on TAMs. Resiquimod, an immune-activating agent, reeducated the TAMs from M2 to M1, boosting the antibody-dependent cellular phagocytosis and enhancing the anti-tumor efficacy [239].

#### Chimeric antigen receptor macrophage (CAR-M) cell therapy

The adoptive cell therapies with chimeric antigen receptor (CAR) technology are getting more and more attention due to the reported efficacy in targeted cancer phagocytosis [240]. CAR-T cell therapy has demonstrated promising efficacy in hematologic malignancies, while its potential application in solid tumors is challenging [240]. Researchers are investigating other immune cells as possible CAR platforms to deal with the limitations of CAR T cells [241, 242]. CAR expression in macrophages plays an essential role in enhancing phagocytosis, polarizing the M2 phenotype to the M1 phenotype, and stimulating the anti-tumor activity of T cells. Folate receptor β (FRβ) is exclusively expressed on TAMs, which is associated with poor clinical outcomes in patients with solid tumors [243]. Conditionally depleting M2-type macrophages with CAR-T cells to target FRβ successfully eliminated the FRβ^+^ M2-TAMs and induced an influx of tumor-specific CD8^+^ T cells [244]. Anti-CD123 CAR-T cells are supposed to provide dual targets for the CD123^+^ tumor cells, and the CD123^+^ M2-type macrophages in the tumor microenvironment efficiently eradicate the Hodgkin’s lymphoma [245].

Morrissey et al. designed new CAR structures named chimeric antigen receptors for phagocytosis (CAR-P). They screened kids of phagocytic receptors in mice, such as FcRɣ, MerTK, and Bai1, and utilized these receptors as the intracellular signaling domains of the CARs. As a result, the CAR-P promoted the phagocytosis of tumor cells and decreased the tumor cell count by over 40% when combined with the Raji B tumor cell line [246]. The anti-CD19 CAR encodes for the intracellular domain of CD3ζ, which is a part of the T cell antigen receptor complex, a type 1 transmembrane protein that forms a homodimer and is involved in ADCP [247].

Other designs of CAR-Ms included tailoring CAR-Ms of target tumors with HER2 overexpression. These CAR-Ms activated the CD147 signaling, thereby inducing matrix metalloproteinases to undermine the extracellular matrix of breast cancer and significantly inhibit tumor growth [248]. CAR-Ms against HER2 lead to a significant decrease in metastatic tumor burden in ovarian cancer. Therefore, CAR-M decreased tumor burden and extended the overall survival in murine models, converted bystander M2 macrophages into M1-type, and boosted anti-tumor T cell activity [240]. CT-0508 has acquired Food and Drug Administration approval as an anti-HER2-CAR-M from CARISMA Therapeutics. The phase I clinical trial of CT-0508 against HER2 positive solid tumors has launched (NCT04660929). Another strategy of CAR-Ms was targeting the CCR7^+^ cells, which were important in inducing cancer cells metastasis to lymphoid organs. CAR-Ms with CCR7 targeting significantly reduced the tumor growth and prolonged the overall survival in a mouse model [249]. Moreover, CAR-Ms generated from induced pluripotent stem cells were capable of activating phagocytosis and reducing the tumor growth in ovarian and pancreatic cancer [250]. The key to the successful clinical use of cell therapy is its scalability and reproducibility. However, CAR-M therapy requires the extraction of the patient's blood and a 1-week manufacturing process, which may be the drawbacks that limit its widespread application.

### TANs-related therapies

**Fig. 4:**
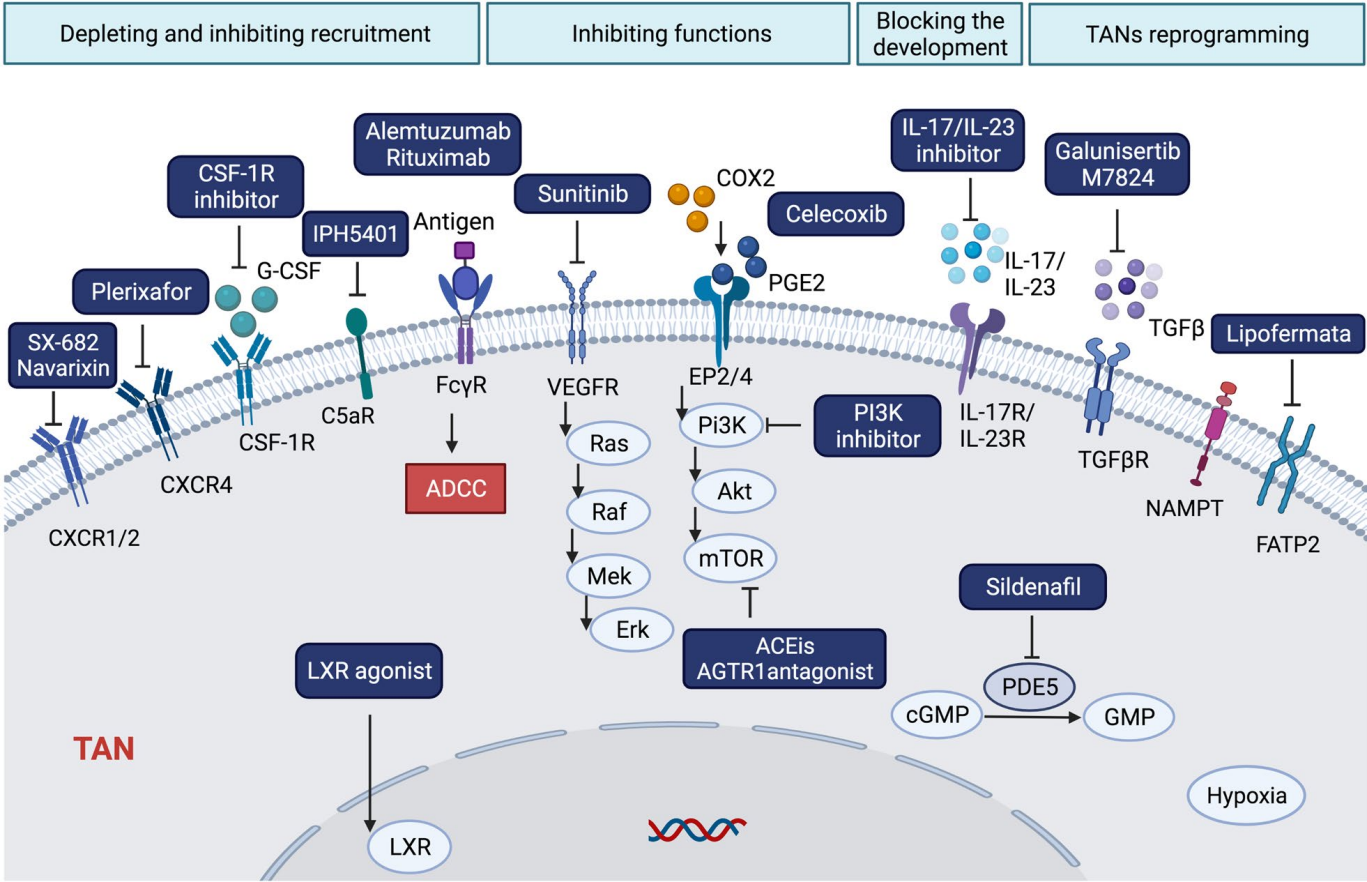
The currently proposed therapy based on TANs targeting. Neutrophil is the first responder to injury. However, the roles and importance of neutrophils in inducing the tumor progression as well as the generation of immunosuppressive microenvironment have been revealed recently. Strategies targeting the TANs to reverse the immunosuppression include depleting and blocking the recruitment of TANs into TME by targeting the CXCR1/2, CXCR4, and CSF-1R, inhibiting the functions of TANs by targeting VEGFR and PGE2, blocking the development of TANs by IL-17/IL-23 inhibitors, and reprogramming TANs by targeting TGF-β, NAMPT, and FATP2

Strategies targeting TANs include depleting TANs and inhibiting their recruitment, inhibiting the functions of TANs, blocking the development of TANs, and reprogramming TANs. We generalized the targeting strategies on TANs for cancer therapy (Fig. 4).

#### Depleting TANs and inhibiting their recruitment

Chemokines CXCL1, 2, 5, 6, 8 and their receptors CXCR1/2 are co-opted by multiple cancers to facilitate the recruitment of neutrophils [251, 252]. A systemic accumulation in CXCR2^+^ TANs correlates with an unfavorable prognosis in patients with pancreatic cancers. CXCR2 blockade prevented the mobilization of neutrophils from circulation and augmented the efficacy of chemotherapies [199]. Thus, the CXCR1/2 inhibitors, SX-682, and navarixin inhibited the extension of TANs and increased the effectiveness of immunotherapy in a mouse model [99].

CXCR4 also contributed to neutrophil recruitment in the tumor metastasis [253, 254]. Deleting CXCR4 in myeloid cells enhanced the anti-tumor immune response, resulting in significantly reduced tumor growth in melanoma [255]. Plerixafor is a CXCR4 antagonist that augments the frequency of circulating neutrophils by releasing them from the marginal pool present in the lungs while preventing neutrophils from returning to the bone marrow. These interactions implicated an essential role of CXCR4-CXCL12 in regulating neutrophil margination in the lungs [256]. Combination therapy with BL-8040/BKT140, a CXCR4 inhibitor, and the pembrolizumab in patients with pancreatic cancer reduced the infiltration of TANs and increased the function of cytotoxic T cells (NCT02826486) [257].

IL-17 cytokine family members, IL-17A and IL-17C, contribute to the recruitment of neutrophils in solid tumors [258–260]. Inhibition of the IL-23 and IL-17 axis decreased the amounts of neutrophils mediated by G-CSF [261, 262]. Hence, inhibition of G-CSF would reduce the number of neutrophils and show anti-tumor efficacy in preclinical tumor models [263]. In addition, the complement component 5-a (C5a) in the TME is also an effective attractant for neutrophils [264, 265]. IPH5401, an anti-C5aR antagonist antibody, is currently being evaluated combined with durvalumab (NCT03665129). Moreover, tumor-derived oxysterols recruit TANs which is dependent on the CXCR2 pathway, thereby facilitating tumor growth by promoting angiogenesis and immunosuppression. Distracting with the oxysterol-CXCR2 pathway delayed the tumor growth and prolonged the overall survival in the tumor-bearing mice model [266].

Neutrophils also expressed FcγRs and eliminated the cancer cells via the antibody-dependent cellular cytotoxicity (ADCC). The depletion of neutrophils directly leads to a reduction in the efficacy of monoclonal antibodies against CD52 (alemtuzumab) or CD20 (rituximab) [267]. Neutrophils in humans are supposed to express FcαRI, also called CD89, a high-affinity receptor for IgA and an inducer of ADCC [268]. FcγR plays an essential role in monoclonal antibody-related anti-tumor therapy. The identity of FcγR-bearing cells that provide cytotoxic activity in tumor patients and the extent to which monoclonal antibodies can manipulate neutrophils remain unknown. The IgA-mediated cytotoxic activity of neutrophils was enhanced after the blockade of the CD47-SIRPα axis [269, 270]. Targeting the CD47-SIRPα axis combined with other therapeutic antibodies increased the anti-tumor efficacy of neutrophils [271, 272].

Neutrophils highly express the ligands of immune checkpoints, including V-domain immunoglobulin suppressor of T cell activation (VISTA) and PD-L1 [273–275]. Blocking VISTA turned the DCs and monocytes into pro-inflammatory cells that promote the activation of T cell-mediated anti-tumor immunity [274]. Blockade of PD-L1 on neutrophils enhanced their cytotoxic activity against tumor cells [276, 277]. Moreover, other checkpoints, such as CD200R, paired immunoglobulin-like type 2 receptor alpha (PILRα), atypical chemokine receptor 2 (ACKR2), leukocyte immunoglobulin-like receptor B2 (LILRB2), and SIRPα, also highly expressed on neutrophils [278–282]. Therefore, depleting TANs and inhibiting their recruitment are an effective preventive approach to prophylactic the immunosuppressive activity of the tumor microenvironment, which may improve the efficacy of immunotherapy.

#### Inhibiting the functions of TANs

Preclinical evidence indicated that the overexpression of COX2 in tumors promotes immune evasion through the production of PGE2. COX inhibition and immune checkpoint inhibitors synergistically promoted the activation of anti-tumor T cells. For advanced melanoma patients, the median time to progression was significantly increased in ICI plus COX inhibition (COXi) versus ICI monotherapy. For patients with NSCLC, ICI plus COXi is also related to prolonged median time to progression compared with ICI alone. In the melanoma and NSCLC cohorts, patients who received ICI plus COXi had a significantly higher objective response rate at 6 months than those who received ICI alone. Hence, compared with ICI alone, in patients with metastatic melanoma and NSCLC, the simultaneous use of COXi and ICI was significantly associated with a longer median time to progression and improved objective response rate [283]. Collectively, these findings identify that the COX inhibition in combination with ICI is a promising target to increase the anti-tumor activity of neutrophils. Other strategies like PI3K inhibitors, PDE5 inhibitors, RTK inhibitors, and sildenafil for inhibiting TANs functions also had positive anti-tumor efficacy similar to the above therapeutic strategy based on MDSCs.

#### Blocking the development of TANs

The recognized IL-12 cytokine family consisted of IL-12, IL-23, IL-27, and IL-35, which play crucial roles in developing immune responses in cancers. Targeting this axis may benefit combination immunotherapies in vivo [284, 285]. Tumor-targeted oncolytic adenovirus would deliver non-secreting IL-12 to tumor cells and significantly enhance the survival of animals with pancreatic cancer [286]. Combination therapy with CAR-T cells targeting tumor-specific EGFRvIII and locally delivered doses of IL-12 achieved effective anti-tumor responses in glioblastoma. Furthermore, IL-12 enhanced the cytotoxicity of CAR-T cells, remodeled the TME, augmented the infiltration of pro-inflammatory CD4^+^ T cells, and reduced the numbers of Tregs [287]. Therapeutic T cells that target tumor antigens are genetically engineered to express membrane-anchored IL-12, which demonstrated limited systemic exposure and improved efficacy in the mouse model [288].

#### Reprogramming TANs

TGF-β is correlated with a poor prognosis in advanced cancers. Aberrant upregulation of TGF-β expression in the TME promoted tumor progression, metastasis, and resistance to immuno-modulatory therapies [289]. Based on this fundamental, several types of TGF-β inhibitors have been evaluated in clinical trials, such as monoclonal, neutralizing, and bifunctional antibodies; antisense oligonucleotides; TGF-β-related vaccines; and receptor kinase inhibitors [290]. The combination of galunisertib, a TGF-β inhibitor, with PD-L1 blockade led to improved inhibition of tumor growth and complete regressions in the colon cancer mouse model [291]. M7824 (MSB0011359C), a bifunctional fusion protein made up of a monoclonal antibody against PD-L1 and the extracellular domain of human TGF-β receptor II, suppressed the tumor growth and metastasis more efficiently than treatment with either anti-PD-L1 antibody or TGF-β trap monotherapy.

Moreover, M7824 is an efficient combination cooperator for chemotherapy or radiotherapy in murine models [292, 293]. Overall, TGF-β is a neutrophil chemotactic agent. Inhibition of TGF-β may affect the recruitment of TANs. Although several clinical trials related to TGF-β inhibitors, their side effects, including off-target effects and cytotoxicity, should be paid attention to. Further studies are needed to understand how manipulation of TGF-β in tumor patients might affect various neutrophil subtypes.

A recent study proved that hypoxia was a potent determinant of TANs phenotypes. Utilizing a uterine cancer mouse model and the administration of respiratory hyperoxia to improve tumor oxygenation. Relief of tumor hypoxia unleashed the tumoricidal potential of TANs through the production of NADPH oxidase-derived reactive oxygen species and MMP-9 [294]. TANs overexpressed FATP2, which was restrained by GM-CSF through the activation of the STAT5 transcription factor. Selectively pharmacological inhibition of FATP2 revoked the suppressive activity of TANs, mediated by arachidonic acid and PGE2, and then substantially delayed tumor progression [172]. The angiotensin-converting enzyme inhibitors and angiotensin II type 1 receptor antagonist attenuated the tumor growth and enhanced the neutrophil hyper-segmentation dependent on the mTOR pathway [295]. Inhibition of nicotinamide phosphoribosyltransferase in TANs led to the anti-tumor conversion of neutrophils and attenuated the tumor angiogenesis and growth in a melanoma mouse model [296].

### Tregs-related therapies

Strategies targeting Tregs include depletion of Tregs, inhibiting the functions of Tregs, and targeting the immune checkpoints on Tregs. Here, we summarized the potential targeted strategies on Tregs for cancer therapy.

#### Depletion of Tregs

Infiltration of Tregs into TME is associated with poor prognosis as the Tregs can diminish anti-tumor immune response. However, conditional depletion of Tregs may concurrently induce the deleterious autoimmunity unless specifically targeting terminally differentiated effector Tregs rather than all Foxp3^+^ T cells [55, 297, 298]. CD25 is a high-affinity receptor subunit of IL-2 and a selective target for Tregs depletion. Tregs depletion enhanced their capacity to elicit strong CD4^+^ conventional T cells responses and ensuing anti-tumor protection [299, 300]. The preclinical assessment of an anti-human CD25 (RG6292) antibody with equivalent characteristics demonstrated efficient Tregs depletion without significant immune-related toxicity [301]. CD25 is expressed at high levels on Tregs and is a target for cancer immunotherapy. Anti-CD25 antibodies exhausted the peripheral Tregs, and the upregulation of inhibitory FcγR IIb at the tumor area prevented the depletion of intra-tumoral Tregs, which may be the reason for the lack of anti-tumor activity previously observed in preclinical models. Fc-optimized anti-CD25 antibody effectively depleted the tumor-infiltrating Tregs, increased effector-to-Treg ratios, improved the control of established tumors, and synergized with anti-PD-1 antibodies to eradicate tumors [302]. Systemic administration of anti-CD25 does not completely eliminate Tregs, but it does prevent Tregs function, thereby safely enhancing tumor immunity in a glioma mouse model [303]. Daclizumab, an anti-CD25 antibody, efficiently depleted all Tregs from the peripheral circulation.

Furthermore, the residual daclizumab suppressed de novo induced Tregs during DC vaccinations [304]. Resistance to PD-1 blockade and radiotherapy is mediated by the upregulation of TIM-3 and the infiltration of Tregs. Treatment with anti-TIM-3 concurrently with anti-PD-L1 and radiotherapy delays tumor growth and decreases Tregs. The analysis of relapsed tumors revealed a resurgence of Tregs. Targeted Tregs depletion with anti-CD25 antibody restored the anti-tumor immunity and lead to tumor rejection and the reaction of immunologic memory [305]. Radiotherapy plus Tregs depletion improved the anti-tumor response. Thus, combining radiotherapy and anti-CD25/CTLA-4 monoclonal antibody resulted in decreased Tregs and enhanced anti-tumor immunity, suppressed the tumor growth, and improved the overall survival (Table 4) [306].

**Table 4:** Ongoing clinical trials to target Tregs in cancer patients

Target	Agent	Tumor types and combinational therapies	Phase	Sample size	Clinical trial number
Anti-CD25 antibody	Daclizumab	Melanoma	I/II	15	NCT00847106
	Basiliximab	Glioblastoma Multiforme	I	34	NCT00626483
Anti- CCR4 antibody	Mogamulizumab (KW-0761)	Advanced Solid Tumors + Nivolumab	I	118	NCT02476123
CCR4 inhibitor	FLX475	Advanced Cancer + Pembrolizumab	I/II	375	NCT03674567
		Gastric Cancer + Pembrolizumab	II	90	NCT04768686
Anti-CTLA-4 antibody	Tremelimumab	Metastatic Urothelial Cancer	II	28	NCT03557918
		NSCLC + Durvalumab	II	15	NCT04625699
		Ovarian Cancer	I/II	50	NCT02571725
		NSCLC + Durvalumab	I	31	NCT03275597
	Ipilimumab	Hepatocellular Carcinoma + Nivolumab	II	40	NCT03510871
		NSCLC + Nivolumab	II	50	NCT03262779
		Renal Cell Carcinoma + Nivolumab	II	74	NCT03297593
		Advanced Melanoma + FLX475	II	20	NCT0489499
Anti-GITR agonistic antibody	BMS-986156	Solid Tumors	I/II	60	NCT04021043
	GWN323	Solid Tumors + PDR001	I	92	NCT02740270
	INCAGN0187	Glioblastoma + INCAGN01876 + Stereotactic Radiosurgery	II	32	NCT04225039
	REGN6569	Squamous Cell Carcinoma of Head and Neck + Cemiplimab	I	85	NCT04465487
	ASP1951	Advanced Solid Tumors + Pembrolizumab	I	436	NCT03799003
Anti-LAG-3 antibody	BMS 986,016 (Relatlimab)	Glioblastoma	I	63	NCT02658981
	Sym022	Solid Tumor	I	15	NCT03489369
	REGN3767	Malignancies	I	669	NCT03005782
	INCAGN02385	Melanoma + INCMGA00012 (anti-PD-1) + INCAGN02390 (anti-TIM-3)	I/II	52	NCT04370704
Anti-LAG-3/PD-L1 antibody	IBI323	Advanced Malignancies	I	322	NCT04916119
	MGD013	Advanced Solid Tumors	I	353	NCT03219268
	RO7247669	Solid Tumors	I	320	NCT04140500
Anti-TIGIT antibody	Ociperlimab (BGB-A1217)	Cervical Cancer + Tislelizumab	II	167	NCT04693234
	AB154	Glioblastoma + AB122	I	46	NCT04656535
	Tiragolumab	NSCLC + Atezolizumab	III	560	NCT04294810
	IBI939	NSCLC	I	42	NCT04672369
Anti-TIGIT/PD-1 antibody	AZD2936	NSCLC	I/II	147	NCT04995523
Anti-OX40 antibody	PF-04518600	Metastatic Renal Cell Carcinoma + Axitinib	II	104	NCT03092856
	MEDI6469	Colorectal Neoplasms	I	4	NCT02559024
	BMS 986,178	Solid Tumors	I	12	NCT03831295
	INCAGN01949	Stage IV Pancreatic and Other Cancers Except Melanoma + CMP-001	I/II	42	NCT04387071
Anti-ICOS antibody	KY1044	Advanced Cancer + Atezolizumab	I/II	412	NCT03829501
	Feladilimab	Head and Neck Squamous Cell Carcinoma + Pembrolizumab	II/III	314	NCT04128696

Treg highly expressed CCR4 which is associated with a poor prognosis in multiple tumors. CCR4 on activated effector Tregs is functionally related to chemical kinetic migration and is responsible for extending these cells to the tumor area [307]. Depletion of Tregs may provide a basis for conducting a clinical trial to study Tregs elimination by administering anti-hCCR4 monoclonal antibody (KM2760) to patients with solid cancer [308]. CCR4 blockade inhibited the tumor growth and the infiltration of Tregs that prolonged the survival in a bladder cancer model [309]. Pharmacological antagonism of the CCR4 receptor with selective CCR4 antagonist, CCR4-35, effectively inhibited the recruitment of Tregs and resulted in enhanced anti-tumor efficacy, overcoming the immune resistance [310]. Depletion of CCR4^+^ T cells is equivalent to total removal of Foxp3^+^ Tregs via CD25^+^ T cells elimination. Administration of anti-CCR4 monoclonal antibody in vivo markedly decreased the Tregs fraction, augmented the responses of NY-ESO-1 specific CD8^+^ T cells, and evoked the anti-tumor immune responses [311]. CCL2 is commonly secreted by macrophages and is essential for recruiting CCR4^+^ Tregs and CCR2^+^ M-MDSCs. Administration of the small-molecule antagonist of CCR4 improved the median survival in a glioblastoma mouse model [312]. Due to the activity of remodeling the TME by targeting CCR4, the immune combinational therapy based on CCR4 targeting is promising. For example, when combined mogamulizumab, an anti-CCR4 antibody, and nivolumab in patients with advanced solid tumors, four out of 15 hepatocellular carcinoma patients (27%) had confirmed tumor responses, one out of 15 pancreatic cancer patients had confirmed response and two out of 15 pancreatic adenocarcinoma patients had unconfirmed responses. Furthermore, during the treatment, the populations of effector Tregs marked by CD4^+^CD45RA^−^FoxP3^high^ reduced, and CD8^+^ T cells in tumor-infiltrating lymphocytes (TILs) augmented (NCT02476123) [313]. A phase I study showed that depletion of Tregs by KW-0761, a humanized CCR4 antibody, was investigated in patients with solid cancer. In this trial, four of 10 patients show stable disease during treatment. In addition, the administration of KW-0761 resulted in the efficient depletion of Foxp3^+^ Tregs in the peripheral blood mononuclear cells. Thus, the combination of KW-0761 with other strategies of immunotherapies, including cancer vaccines or ICIs, is a promising approach to enhance the anti-tumor immune responses [314].

#### Inhibiting the functions of Tregs

The maintenance and function of Tregs are dependent on PI3Kδ signaling. Selectively depletion of Tregs with PI3K inhibitors increased the activity of CD8^+^ T cells and prevented tumor metastasis [315–317]. Besides, impairment of the activation of PI3K pathway through silencing the microRNA-126 in a breast cancer model reduced the induction and suppressive function of Tregs [318]. Moreover, inhibition of the PI3K activity combined with anti-PD-1 and/or anti-CTLA-4 monoclonal antibodies enhanced the therapeutic efficacy in mouse models of head and neck cancer [319]. Therefore, these findings suggested that inhibiting PI3K signaling combined with ICIs can selectively deplete Tregs, reduce the suppressive function of Tregs, and alleviate Treg-mediated resistance.

Tregs secreted cytokine IL-10, which is associated with poor prognosis in cancers, involving the activation of tumor-resident APC and suppression of effective T cell functions [320, 321]. Moreover, IL-10^+^ and IL35^+^ Tregs cooperatively limited the anti-tumor immune response by Blimp-1-mediated exhaustion of tumor-infiltrating T cells and upregulation of immune checkpoints, including PD-1, TIM-3, lymphocyte activation gene 3 (LAG-3), T cell immunoglobulin and immunoreceptor tyrosine-based inhibitory motif domain (TIGIT) [322]. Furthermore, IL-10 deficiency reduced the Tregs-mediated immunosuppression through reduction of the expression of neuropilin on Tregs, leading to the tumor regression and enhancing the anti-tumor immunity [323, 324]. The pegylated recombinant human IL-10, AM0010 or pegilodecakin, increased the serum levels of IFN-γ and IL-18 and reduced the levels of TGF-β. These alterations of cytokines resulted in activated intra-tumoral CD8^+^ T cells and induced the objective tumor responses in advanced solid tumors (NCT02009449) [325, 326]. Furthermore, the combination of AM0010 with PD-1 blockade induced the expansion of LAG-3^+^ PD-1^+^ CD8^+^ T cells [326]. Besides, blocking IL-35 increased the number of tumor-infiltrating effective T cells, enhancing the anti-tumor immune response and reducing tumor growth in multiple mouse models [327].

CD39/CD73/Adenosine receptor (A2AR) pathways are potential targets to reduce the induction and suppressive function of Tregs [328–331]. A2AR stimulation enhanced the Treg-mediated immunosuppressive mechanism functionally. In addition, A2AR-mediated stimulation of lymphocytes using A2AR agonists induced the expansion of Tregs ex vivo [332]. Moreover, CGS21680, an A2AR agonist, upregulated the expression of CD39 and CD73 through E2F transcription factor 1 and cyclic adenosine monophosphate response element-binding protein in Tregs [333]. Besides, utilizing the AR antagonists and antibodies to target the adenosinergic pathway in preclinical mice models had favorable anti-tumor immune responses and would further enhance the sensitivity of tumors to ICIs therapies in acquired resistance cases [334–337]. Furthermore, inhibiting the adenosine/A2AR signaling pathway enhanced the efficacy of CAR T cell transfer. Thus, combining an A2AR inhibitor with CAR T cell therapy has shown therapeutic efficacy in preclinical models [338, 339].

#### Targeting the immune checkpoints on Tregs

Tregs are also with upregulated expression of immune checkpoints. Targeting the immune checkpoints on Treg cells is proposed to be effective for cancers [340–342]. For example, CTLA-4 is highly expressed on Treg cells as a co-inhibitory receptor [343]. However, CD47 expression on Tregs is supposed to limit the anti-CTLA-4-mediated elimination. Simultaneously adjusting the “eat me” and “don't eat me” signals leads to the consumption of Tregs in the TME, which may be an efficient strategy for the treatment of solid tumors. Dual targeting of CTLA-4 and CD47 on Tregs preferentially exhausted ICOS^high^ immunosuppressive Treg cells in the TME and boosted immunity to solid tumors [344]. Tregs restrained the T cell stimulatory activity of APCs by reducing the expression of CD80/CD86 by trogocytosis depending on CTLA-4. The reduction of CD80/CD86 on APCs exerted double suppressive effects on T cell-mediated immune responses by restraining CD80/CD86 co-stimulation to naïve T cells and improving free PD-L1 available on effector T cells. Combination with the blockade of CTLA-4 and PD-1/PD-L1 synergistically inhibited the Tregs-mediated immune suppression and then enhanced the immune responses [345, 346]. The blockade of CTLA-4 triggered CD28-dependent hyper-proliferation of Tregs in the TME, and the inactivation of accompanied Tregs is required to achieve tumor regression. Tregs self-regulate through a feedback loop that relies on CTLA-4 and CD28, which adjusts their accounts based on the amount of local co-stimulation [347–349]. Therefore, the disruption by CTLA-4 blockade may provide cancer patients with therapeutic benefits.

Glucocorticoid-induced tumor-necrosis-factor receptor (TNFR)-related protein (GITR, also known as TNFRSF18) is a potential marker of Treg cells. The co-stimulation of GITR-specific antibodies and other co-stimulatory molecules (CD28 or 4-1BB, also known as CD137, or OX40, also known as CD134) on responder T cells function always dominates the co-stimulatory inhibitory functions of Tregs [350, 351]. The combination of anti-TIM-3 ligand galectin-9 and an agonistic GITR antibody depleting Tregs induced the synergistic anti-tumor activity [352]. MK-4166, an anti-human GITR antibody, decreased the numbers of CD44^hi^ICOS^hi^ intra-tumoral Tregs and immune suppressor phenotype while enhancing the effector responsiveness. Intra-tumoral CD8^+^ T cells acquire a more functional phenotype featured by downregulation of the exhaustion markers PD-1 and LAG-3 [353]. The phase I trial of an anti-GITR antibody, TRX518 (NCT01239134) monotherapy in patients with advanced cancer, demonstrated that TRX518 reduced the circulating and intra-tumoral Tregs. However, the revival of T cells with PD-L1 blockade overwhelms the resistance of advanced cancers to anti-GITR monotherapy. Based on this evidence, the clinical trial using TRX518 and PD-1 blockade in patients with advanced/refractory solid tumors started (NCT02628574) [354]. Treatment with adenovirus-based vaccine plus N-803, OX40, GITR, and IDO inhibitor resulted in decreased immunosuppression of Tregs in the TME, and then the inhibition of tumor growth and protection from cancer cells rechallenge in 4T1 and LL2 models. Monotherapy with each of these agents had limited anti-tumor efficacy. However, the combination of these agents has increased anti-tumor activity, which provides the rationale for the combination of multi-modal immunotherapies to enable adaptive anti-tumor immunity [355].

Tregs express LAG-3, a CD4-associated molecule that binds to MHC-II, contributing to Tregs suppressor activity. LAG-3 antibodies inhibited the suppression by induced Treg cells both in vitro and in vivo [356]. Antibody-mediated elimination of 4-1BB-expressing Tregs decreased the tumor growth without negatively affecting the function of CD8^+^ T cells representing a strategy with potential anti-tumor activity [357]. When comparing anti-tumor activity between the anti-4-1BB/anti-PD-1 and the anti-PD-1/anti-LAG-3 in the B16-F10 melanoma mouse model, tumor regression occurred in animals receiving anti-PD-1 anti-4-1BB was more concomitantly [358].

TIGIT is also over-expressed on Treg cells and associated with immunosuppression [359, 360]. Anti-TIGIT/CD155 pathway treatment significantly inhibited tumor growth in mouse models of transgenic head and neck squamous cell carcinoma. It improved the anti-tumor immune responses by activating the function of CD8^+^ T cells and reducing the Tregs [361]. OX40 is expressed with higher levels in murine and human Tregs, promoting Tregs' development and proliferation [362, 363]. Anti-OX40 monoclonal antibody therapies indicated that CD8^+^ T cell expansion or Tregs consumption might be preferred according to the composition of different cancers [364]. ATOR-1015, a human CTLA-4 and OX40 bi-specific antibody, induced the activation of T cells and depletion of Treg cells, then reduced the tumor growth, and improved the survival in several syngeneic tumor models. ATOR-1015 is expected to have a synergistic effect when combined with anti-PD-1/PD-L1 therapies. Preclinical data suggested that the further clinical application of ATOR-1015 and the first-in-human trial have been launched (NCT03782467) [365]. Combining anti-OX40 and anti-CTLA-4 immunotherapy boosted tumor regression and tumor-bearing host survival depending on CD4^+^ and CD8^+^ T cells [366].

Inducible T cell co-stimulator (ICOS) is a highly inhibitory antigen-specific T cell marker with characteristics of Th17/Th1 and Treg cells [367]. ICOS-legend expression by tumor cells directly drove the activation and expansion of Treg in TME [368]. High-dose IL-2 therapy increased the expansion of the ICOS^+^ Treg population, which may predict clinical outcomes in melanoma [369]. F42K, a mutant IL-2, induced a systematic decrease in the expansion of ICOS^+^ Treg cells, which promoted the expansion of NK cells and inhibited the tumor growth of melanoma more efficiently than wild-type IL-2 [370]. Akt-mTOR pathway is an essential negative regulator of the differentiation and expansion of Tregs. TCR and IL-2 signals provide the significant inputs for mTORC1 activation, which program the suppressive function of Tregs [371, 372]. KY1044, a fully human IgG1 antibody bounding to ICOS, induced sustained depletion of ICOS^+^ Treg cells and increased the intra-tumoral Teff/Treg ratio. This KY1044 monotherapy has been demonstrated to improve the anti-PD-L1 efficacy in cancers [373]. Tregs-targeted therapy combined with ICIs can attenuate Treg-mediated drug resistance and increase tumor cell sensitivity to treatment, thereby promoting tumor regression. Several clinical trials are ongoing to investigate the effects of small molecule inhibitors, monoclonal antibodies, multiple ICIs, and combined therapeutics (Table 4).

### tDCs-related therapies

#### DC vaccination

DC vaccines induced an enhanced anti-tumor immune response against tumor antigens. Currently, many clinical trials are designed and ongoing with various DC subsets [374, 375]. For example, a phase I/II study evaluated 10 ovarian cancer patients who received autologous monocyte-derived DC and IL-2 after initial debulking and chemotherapy. Three out of 10 patients showed a sustained complete response, and two patients achieved a partial response. In patients with long-term survival, the improved immunologic characteristics, including increased activity of NK cells, increased IFN-γ-secreting T cells, immunostimulatory cytokine secretion, and decreased secretion of immunosuppressive factors, were observed after DC vaccination [376]. A phase I study (NCT02285413) involved patients with stage III/IV melanoma who received autologous monocyte-derived DC vaccination. The antigen-specific CD8^+^ T cells were found in 44% versus 67%, T cell responses in 28% versus 19% in patients who received DC vaccination with and without cisplatin. Combination therapy with DC vaccination and cisplatin in melanoma resulted in more tumor-specific T cell responses than the monotherapy of DC vaccination [377]. A phase I study resembling a viral infection in tumor tissue combined with a DC vaccine and ablative radiotherapy showed immune-associated activity and preliminary clinical therapeutic effect signs. Nine out of 15 pre-treated patients with castration-resistant prostate cancer presented stable disease [378]. Gene and protein expression analyses in lymphocytes and cancer samples after DC vaccination identified vital immunoregulatory pathways, including PD-1 and CTLA-4, which correlated with inferior clinical outcomes [379]. In addition, a phase I trial (NCT01946373) investigated the safety/feasibility of TILs adoptive cell therapy combined with DC vaccination in patients with melanoma progressing after ICIs. Two out of 5 patients experienced complete responses. Two patients had partial responses [380]. A preclinical study has shown the promising efficacy of DC vaccination in combination with CD40-stimulation for pancreatic cancer and deserves future clinical trials [381]. Despite the success of DC vaccines in multiple preclinical studies and clinical trials, there are many challenges associated with them, including the lack of a universal vaccine, the high cost of treatment, the need for a large number of DCs, and the difficulty of isolating cDCs.

#### Reprogramming tDCs to be immunostimulatory with nanoparticles

Isolating and reprogramming DCs to induce tolerogenic DCs are a novel immunotherapy strategy for cancers. Nanomedicines targeting DCs to perform this reprogramming process induced comprehensive immuno-modulations [382]. Lipid nanoparticle delivery of siRNAs with a single-chain antibody to target DCs restricted their uptake and conferred profound gene knockdown to restrain robust immune responses [383]. Currently, modification of adjuvant imiquimod (R837) plus immunomodulator 1-methyl-d-tryptophan (1 MT)@ZIF-8 with mannan to achieve DCs-targeted immune amplification, which adequately activated the immune response and supplied effective platform against advanced malignancies [384]. In addition, nanoparticles display that anti-DC-specific intracellular adhesion molecule-3-grabbing non-integrin (DC-SIGN; CD209) antibody loaded with rapamycin (sirolimus) is favorable to modify DCs. Rapamycin-loaded DC-SIGN nanoparticles' uptake efficiency into DCs resulted in a maturation resistant phenotype and inhibited the extend of allogeneic T cells [385].

In conclusion, reprogrammed tDCs with nanoparticles can elicit enhanced anti-tumor immunity, which may serve as a novel therapeutic strategy to suppress malignant cell survival. Nevertheless, there are still some problems that limit the application of nanoparticles. For example, the persistence of nanoparticle-induced immune tolerance may involve multiple nanoparticle administrations; the long-term impact of nanoparticle safety and side effects needs to be further assessed; there may be difficulties with the manufacture and regulatory approval of complexed nanoparticles.

## Conclusions and perspectives

With the development of immunotherapies, the critical roles of immunosuppressive during cancer immunotherapies have been revealed. The efficacy of targeting these immunosuppressive cells to increase the effectiveness of immunotherapies or reverse the resistance of immunotherapies is promising and encouraging. However, the infiltration of these immunosuppressive cells is associated with cancer types, stages, and the adopted therapies. Thus, individually analyzing the TME of advanced tumors, identifying the phenotype of these immunosuppressive cells in TME is critical for developing related treatment. In addition, the generation, expansion, recruitment, and activation of immunosuppressive cells in the tumor microenvironment involve complex mechanisms, making it difficult for a single strategy to target any one of these cells to induce robust anti-tumor effects [386]. Therefore, immunosuppressive cell-targeted therapy combined with other anti-tumor therapies is the preferred strategy. At this time, the dose, timing, and sequence of treatment should be fully considered. Besides, totally deleting these cells may cause universal loss of innate immune for the overlap targets between immunosuppressive cells in TME and the organ resident innate immune cells. The solution must be a complex combination; in addition to supporting the inflammatory phenotype of immune cells, it also includes the selective suppression or consumption of powerful tumor suppressor cytokines and cell types.

Second, there have overlaps of targets among different immunosuppressive cells, like M-MDSCs and M2-TAMs. Thus, some dual targets are proposed to have better efficacy, like CSF-1R. For the engineered CAR-M, which is still at its nascent stage with limited clinical trials initiated, the limitations of CAR-M have yet to be unfolded. For Tregs targeting, the therapies are predominately focused on the ICIs that are expressed on Tregs. tDCs-related treatments mainly focused on DC-related vaccines to modulate the TME. The process of establishing clinically applicable DC vaccines is complex and with high technical requirements. Another issue needed to emphasize is the sequence of administration of these therapies based on immunosuppressive cell, and the efficacy based on these therapies largely depends on the availability of appropriate drug delivery strategies. Besides, suspension of treatment may lead to recovery of the TME and increase disease progression. Overall, targeting immunosuppressive cells has shown synergistic anti-tumor efficacy with immunotherapies. However, the further mechanisms of these cells participating in the tumor immune response and the optimization of clinical application of therapies targeting these cells are needed.

## Data Availability

The materials supporting our conclusion of this review are included within the article.

## Abbreviations

MDSCs: Myeloid-derived suppressive cells
TAMs: Tumor-associated macrophages
TANs: Tumor-associated neutrophils
Tregs: Regulatory T cells
tDCs: Tumor-associated dendritic cells
ICIs: Immune checkpoint inhibitors
TME: Tumor microenvironment
VEGF: Vascular endothelial growth factor
TGF-β: Transforming growth factor beta
IL-10: Interleukin-10
EMT: Epithelial–mesenchymal transition
MMPs: Matrix metalloproteinases
NETs: Neutrophil extracellular traps
PGE2: Prostaglandin E2
TIM-3: T cell immunoglobulin and mucin domain 3
5-FU: 5-Fluorouracil
BTK: Bruton's tyrosine kinase
NLRP3: Nucleotide oligomerization domain-like receptor family pyrin domain-containing 3
PDE5: Phosphodiesterase-5
HDAC: Histone deacetylase inhibitor
Nrf2: Nuclear factor erythroid 2-related factor 2
ATRA: All-trans-retinoic acid
CK2: Casein kinase 2
LXR: Liver-X nuclear receptor
CTL: Cytotoxic T lymphocyte
ApoE: Apolipoprotein E
LRP: Lipoprotein receptor-related protein
FATP2: Fatty acid transport protein 2
IDO: Indoleamine 2,3-dioxygenase
SIRPα: Signal regulatory protein alpha
Siglec-10: Sialic-acid-binding Ig-like lectin 10
ADCP: Antibody-dependent cellular phagocytosis
CAR: Chimeric antigen receptor
FRβ: Folate receptor β
ADCC: Antibody dependent cellular cytotoxicity
VISTA: V-domain immunoglobulin suppressor of T cell activation
TCR: T cell antigen receptor
TTP: Time to progression
ORR: Objective response rate
TILs: Tumor-infiltrating lymphocytes
LAG-3: Lymphocyte activation gene 3
TIGIT: T cell immunoglobulin and immunoreceptor tyrosine-based inhibitory motif domain
TNFR: Tumor-necrosis-factor receptor
GITR: Glucocorticoid-induced tumor-necrosis-factor receptor;
ICOS: Inducible T cell co-stimulator
